# Supplementary material for: K13-Mediated Reduced Susceptibility to Artemisinin in Plasmodium falciparum Is Overlaid on a Trait of Enhanced DNA Damage Repair
Source: Cell Rep. 2020 Aug 4;32(5):107996. doi: 10.1016/j.celrep.2020.107996 (PMC7408483; doi:10.1016/j.celrep.2020.107996)
Supplement: Document S1. Figures S1–S5 and Tables S1–S3 [file mmc1.pdf]

Cell Reports, Volume *32*

Supplemental Information

K13-Mediated Reduced Susceptibility to Artemisinin in *Plasmodium falciparum* Is Overlaid on a Trait of Enhanced DNA Damage Repair

Aoli Xiong, Prem Prakash, Xiaohong Gao, Marvin Chew, Ian Jun Jie Tay, Charles J. Woodrow, Bevin P. Engelward, Jongyoon Han, and Peter R. Preiser

Supplemental material

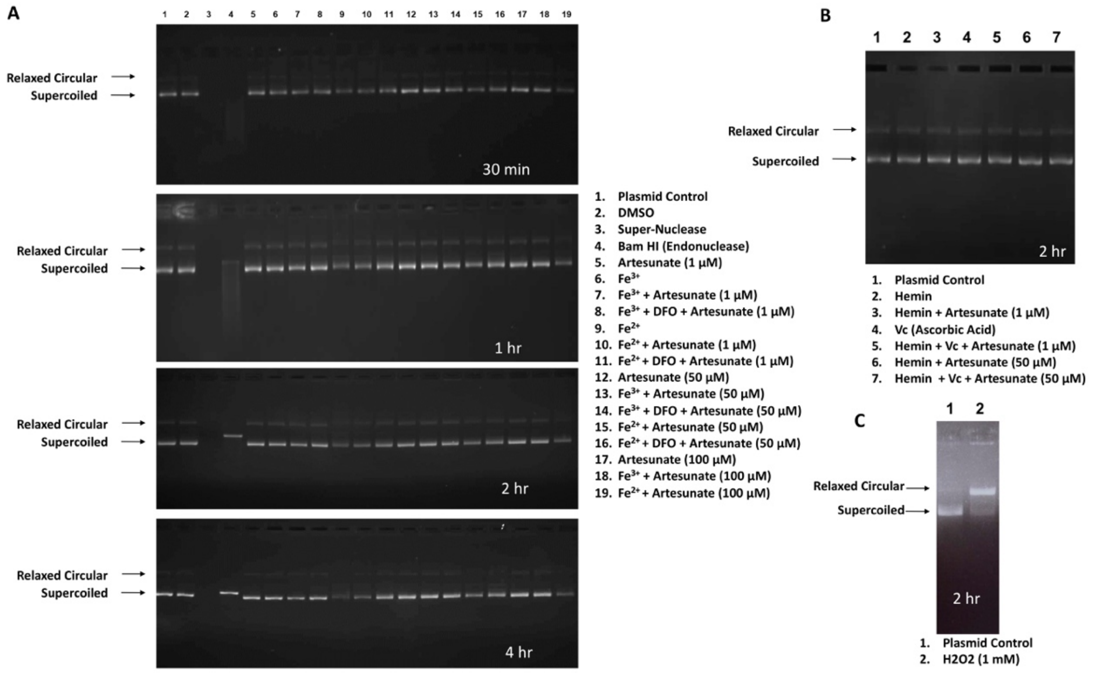

Supplementary figure 1. Activated artesunate does not induce DNA damage of purified DNA
A. Induced activation of artesunate by ferrous ion (Fe^{2+}) and its effect on purified plasmid DNA. B. Activation of artesunate by reduced hemin (haem) using ascorbic acid (Vc) and plasmid treatment. C. H_2O_2 used as positive control for plasmid DNA damage. Related to STAR Method.

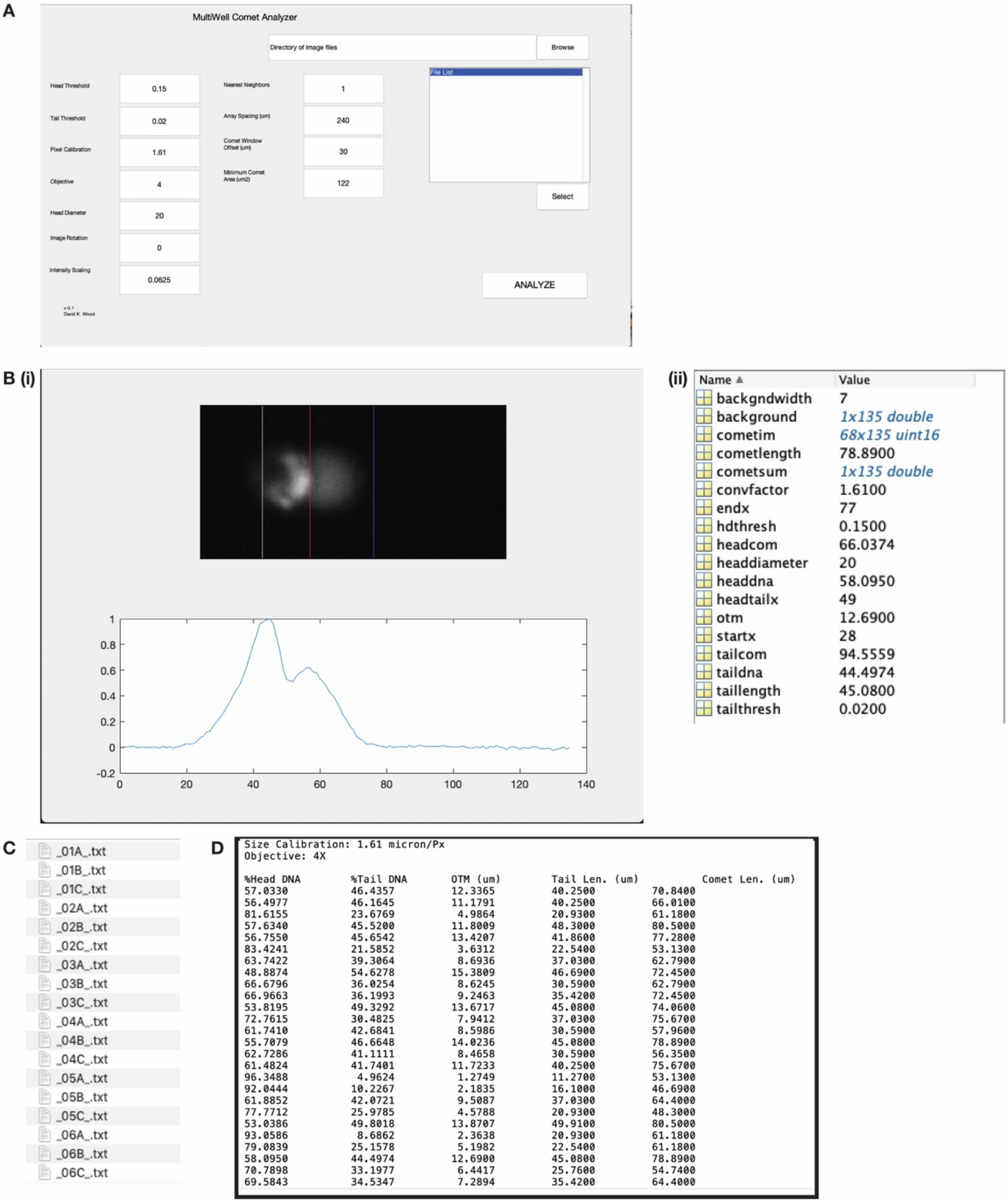

Size Calibration: 1.61 micron/Px
Objective: 4X

%Head DNA	%Tail DNA	OTM (um)	Tail Len. (um)	Comet Len. (um)
57.0330	46.4357	12.3365	40.2500	70.8400
56.4977	46.1645	11.1791	40.2500	66.0100
81.6155	23.6769	4.9864	20.9300	61.1800
57.6340	45.5200	11.8009	48.3000	80.5000
56.7550	45.6542	13.4207	41.8600	77.2800
83.4241	21.5852	3.6312	22.5400	53.1300
63.7422	39.3064	8.6936	37.0300	62.7900
48.8874	54.6278	15.3809	46.6900	72.4500
66.6796	36.0254	8.6245	30.5900	62.7900
66.9663	36.1993	9.2463	35.4200	72.4500
53.8195	49.3292	13.6717	45.0800	74.0600
72.7615	30.4825	7.9412	37.0300	75.6700
61.7410	42.6841	8.5986	30.5900	57.9600
55.7079	46.6648	14.0236	45.0800	78.8900
62.7286	41.1111	8.4658	30.5900	56.3500
61.4824	41.7401	11.7233	40.2500	75.6700
96.3488	4.9624	1.2749	11.2700	53.1300
92.0444	10.2267	2.1835	16.1000	46.6900
61.8852	42.0721	9.5087	37.0300	64.4000
77.7712	25.9785	4.5788	20.9300	48.3000
53.0386	49.8018	13.8707	49.9100	80.5000
93.0586	8.6862	2.3638	20.9300	61.1800
79.0839	25.1578	5.1982	22.5400	61.1800
58.0950	44.4974	12.6900	45.0800	78.8900
70.7898	33.1977	6.4417	25.7600	54.7400
69.5843	34.5347	7.2894	35.4200	64.4000

Supplementary figure 2. Illustration of image analysis using guicometanalyzer algorithm. Guicometanalyzer is a customized software developed in MATLAB that used to analyze images generated from CometChip experiment. A. Dialogue window for file selection and parameter setting. When running the software in MATLAB, the dialogue window would pop out, where parameters can be set or adjusted. Image files, labelled using the well position (i.e. 01A), can be then imported by browsing file direction. B. Interim result of individual comet. (i) Comet head (between while line and red line) and tail (between red line and blue line) recognized by the algorithm. (ii) Values reported by the algorithm. During the analysis, the algorithm can automatically identify comet, differentiate head and tail and report values accordingly as shown in the figure. C. Text file list generated by the algorithm for each macro-well. D. Data layout in each text file. For each image file representing one macro-well, a text file containing all the reported value will be generated. Median can then be calculated for each well and exported to excel file for further analysis. Related to STAR Method.

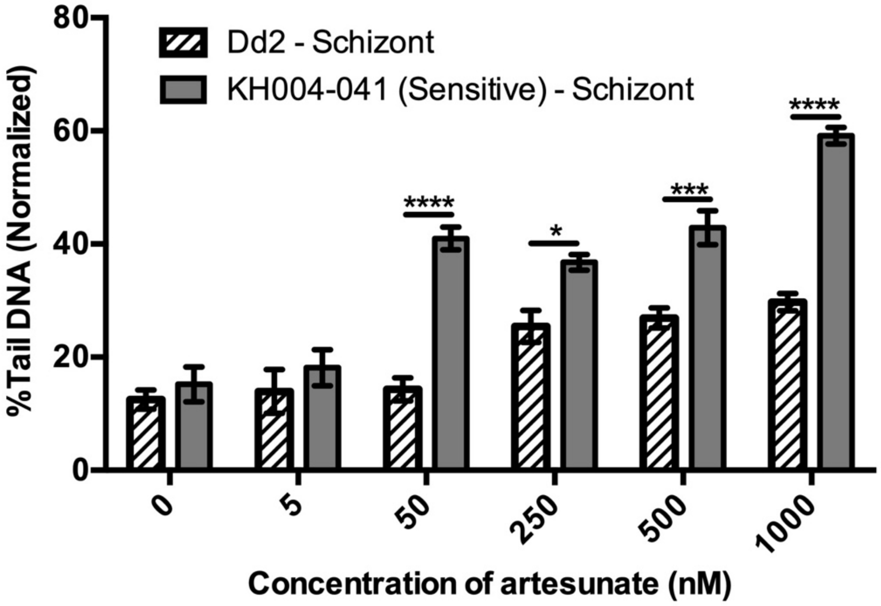

Supplementary figure 3. Comparison of artesunate-induced DNA damage level in Dd2 and Sensitive Cambodian isolate KH004-041.

Schizont stage parasites were treated with different concentration of artesunate for 1 hour and subjected to analysis on alkaline CometChip. Data was normalized within each treatment group. N≥3. *p<0.05, ***p<0.001, ****p<0.0001, two-way ANOVA. Comparison were made between Dd2 and KH004-041 at each concentration. Same data were plotted separately in Figure 2B and Figure 3A. Related to Figure 2 and Figure 3.

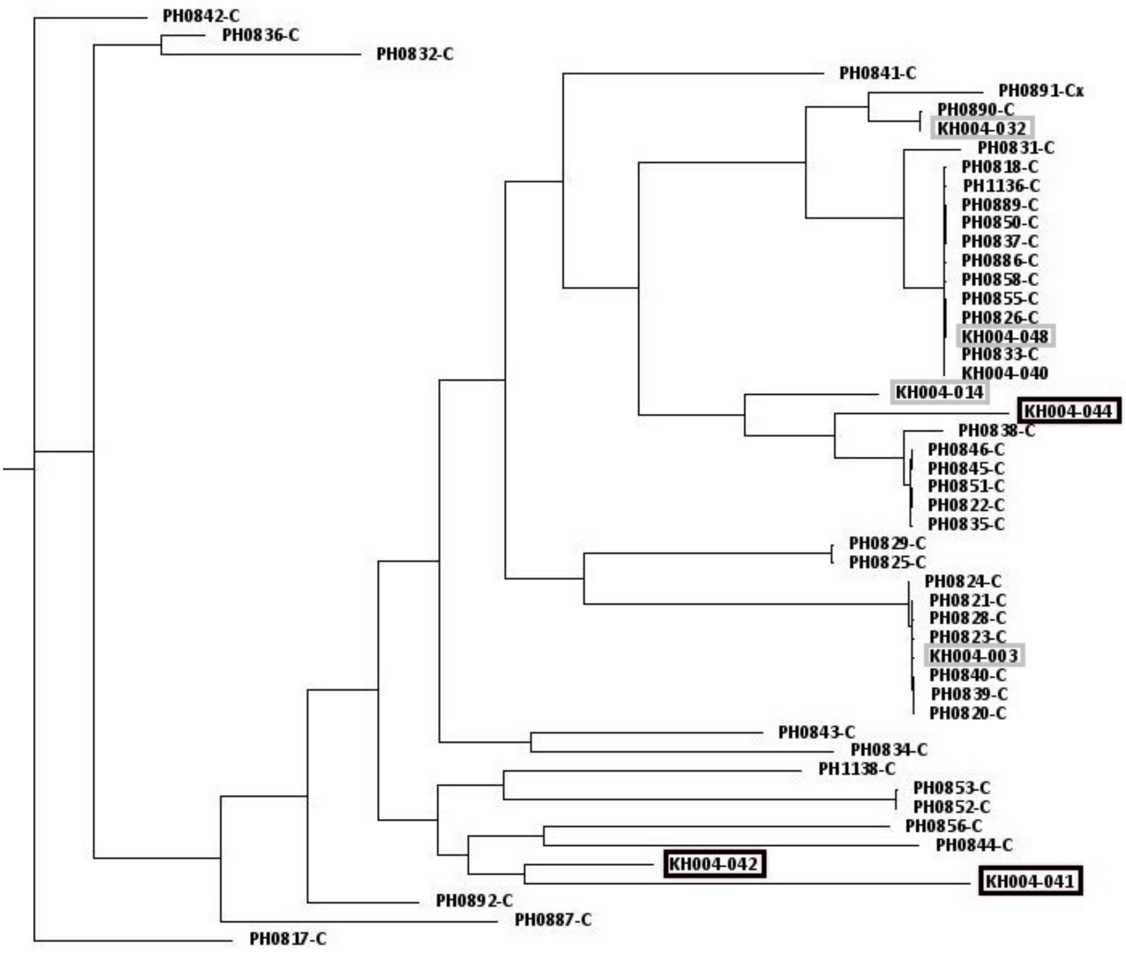

Supplementary figure 4. Phylogenetic tree based on SNP profile of samples from Pailin, Cambodia 2011.
VCF file containing SNPs across the whole genome were obtained from open source Pf3K project release 5.1 and analyzed as described in the Method. Black and grey box highlighted strains tested using CometChip. Among them, artemisinin sensitive isolates are labeled in black box, and artemisinin resistant isolates are labeled in grey box. Related to Figure 3 and STAR Method.

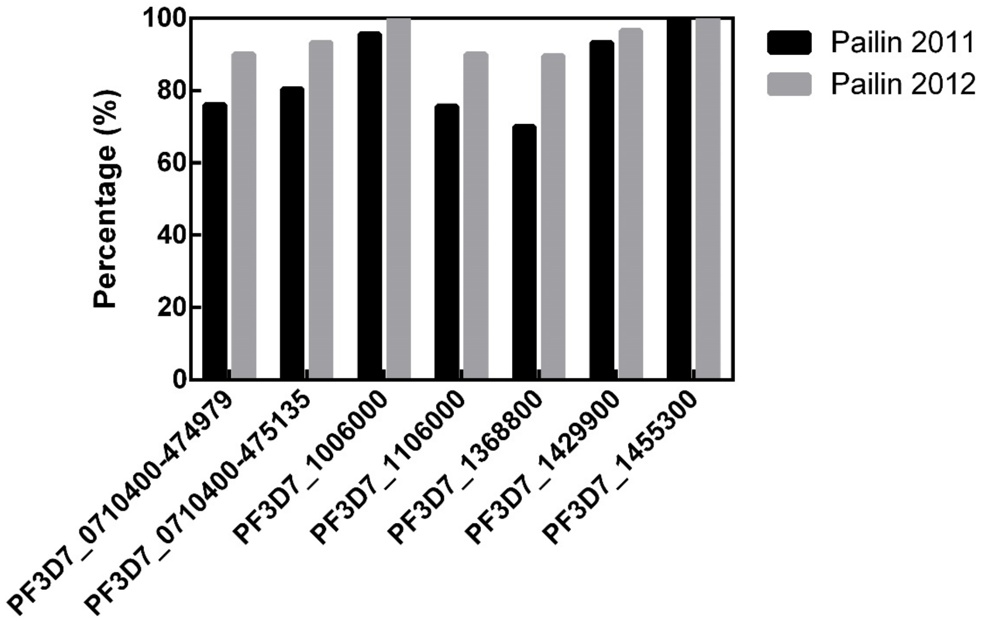

Supplementary figure 5. Temporal change of the frequency of the 'EnDNArep genotype' in K13 parasites from 2011 (black bar) to 2012 (grey bar) in Pailin, Cambodia.

Related to Figure 5.

Supplementary table 1. Distribution of mutations in 'EnDNArep genotype' in the KH and Dd2 strains. Artemisinin sensitive (red) and resistant (black) strains were compared. Mutant, heterozygote and wild type were labeled in orange, yellow and white, respectively. Related to table 1

Gene ID	Position	KH004-041	KH004-042	Dd2	KH004-044	KH004-003	KH004-014	KH004-040	KH004-048	KH004-032
PF3D7_0710400	474979									
	475135									
PF3D7_1006000	257175									
PF3D7_1106000	257027									
PF3D7_1368800	2731444									
PF3D7_1429900	1178944			TGA TAA TG	*	*	T	T	T	*
PF3D7_1455300	2260945									

Wild type

Heterozygote

Mutant

Supplementary table 2. List of affected genes. Related to Table 1.

Gene ID	Position	Product	ID of Affected gene(s)	Product description	Predicted function of affected gene(s)
PF3D7_0710400	474979 & 475135	DNA repair protein RAD14, putative	PF3D7_0710200	conserved Plasmodium protein, unknown function	GO:0005634 nucleus
			PF3D7_0710300	conserved protein, unknown function	GO:0016021 integral component of membrane; GO:0006810 transport; GO:0020011 apicoplast
			PF3D7_0710500	conserved protein, unknown function	GO:0020011 apicoplast
			PF3D7_0710600	60S ribosomal protein L34	GO:0005622 intracellular; GO:0005840 ribosome; GO:0003735 structural constituent of ribosome; GO:0006412 translation; GO:0022625 cytosolic large ribosomal subunit; GO:0005634 nucleus
PF3D7_1006000	257175	IMP1-like protein, putative	PF3D7_1005800	conserved Plasmodium membrane protein, unknown function	GO:0016021 integral component of membrane; GO:0005524 ATP binding; GO:0006810 transport
			PF3D7_1005900	conserved protein, unknown function	GO:0005737 cytoplasm; GO:0005524 ATP binding; GO:0016787 hydrolase activity; GO:0016740 transferase activity; GO:0020011 apicoplast
			PF3D7_1006100	CCR4-NOT transcription complex subunit 5, putative	GO:0005634 nucleus; GO:0006355 regulation of transcription, DNA-templated; GO:0030014 CCR4-NOT complex
			PF3D7_1006200	DNA/RNA-binding protein Alba 3	GO:0003676 nucleic acid binding; GO:1903561 extracellular vesicle; GO:0020020 food vacuole; GO:0005634 nucleus; GO:0003677 DNA binding; GO:0003723RNA binding; GO:0003729 mRNA binding; GO:0005515 protein binding
PF3D7_1106000	257027	RuvB-like helicase 2	PF3D7_1105800	conserved Apicomplexan protein, unknown function	GO:0020020 food vacuole; GO:0005739 mitochondrion; GO:0003729 mRNA binding
			PF3D7_1105900	conserved Plasmodium membrane protein, unknown function	GO:0016021 integral component of membrane; GO:0005886 plasma membrane; GO:0006810 transport

			PF3D7_1106100	apicoplast ribosomal protein S15 precursor, putative	GO:0005622 intracellular; GO:0005840 ribosome; GO:0003735 structural constituent of ribosome; GO:0006412 translation; GO:0020011 apicoplast
			PF3D7_1106200	conserved Plasmodium protein, unknown function	GO:0020011 apicoplast
PF3D7_1368800	2731444	DNA repair endonuclease XPF, putative	PF3D7_1368700	mitochondrial carrier protein, putative	GO:0022857 transmembrane transporter activity; GO:0055085 transmembrane transport; GO:0005743 mitochondrial inner membrane; GO:0006810 transport
PF3D7_1429900	1178944	ADP-dependent DNA helicase RecQ	PF3D7_1429800	coatomer subunit beta, putative	GO:0030126 COPI vesicle coat; GO:0030117 membrane coat GO:0005488 binding; GO:0005198 structural molecule activity; GO:0006886 intracellular protein transport; GO:0016192 vesicle-mediated transport; GO:0006888ER to Golgi vesicle-mediated transport
			PF3D7_1430000	conserved protein, unknown function	GO:0005737cytoplasm; GO:0016020 membrane
PF3D7_1455300	2260945	conserved Plasmodium protein	PF3D7_1455200	methyltransferase, putative, unspecified product	GO:0008168 methyltransferase activity; GO:0009234 menaquinone biosynthetic process; GO:0008152 metabolic process; GO:0006744 ubiquinone biosynthetic process
			PF3D7_1455400	hemolysin III	GO:0016021 integral component of membrane; GO:0020020 food vacuole; GO:0016020 membrane; GO:0044179 hemolysis in other organism

Supplementary table 3. List of known DNA repair genes. Related to Table 1.

Gene ID	Gene group	Gene function
PF3D7_0107800	GO	GO-0006281-BP-DNA repair
PF3D7_0107800	GO	GO-0006302-BP-double-strand break repair
PF3D7_0203300	GO	GO-0006289-BP-nucleotide-excision repair
PF3D7_0206000	GO	GO-0006281-BP-DNA repair
PF3D7_0217400	GO	GO-0006281-BP-DNA repair
PF3D7_0305600	GO	GO-0006281-BP-DNA repair
PF3D7_0408500	GO	GO-0006281-BP-DNA repair
PF3D7_0416400	GO	GO-0006281-BP-DNA repair
PF3D7_0505500	GO	GO-0006281-BP-DNA repair
PF3D7_0505500	GO	GO-0006298-BP-mismatch repair
PF3D7_0509500	GO	GO-0006281-BP-DNA repair
PF3D7_0513600	GO	GO-0006281-BP-DNA repair
PF3D7_0514100	GO	GO-0006281-BP-DNA repair
PF3D7_0605800	GO	GO-0006302-BP-double-strand break repair
PF3D7_0614800	GO	GO-0006284-BP-base-excision repair
PF3D7_0614800	GO	GO-0006281-BP-DNA repair
PF3D7_0619100	GO	GO-0006281-BP-DNA repair
PF3D7_0619100	GO	GO-0009380-CC-excinuclease repair complex
PF3D7_0628600	GO	GO-0006281-BP-DNA repair
PF3D7_0706700	GO	GO-0006298-BP-mismatch repair
PF3D7_0710100	GO	GO-0006281-BP-DNA repair
PF3D7_0710400	GO	GO-0006289-BP-nucleotide-excision repair
PF3D7_0725000	GO	GO-0006298-BP-mismatch repair
PF3D7_0726300	GO	GO-0006298-BP-mismatch repair
PF3D7_0803400	GO	GO-0000724-BP-double-strand break repair via homologous recombination
PF3D7_0910500	GO	GO-0006281-BP-DNA repair
PF3D7_0917100	GO	GO-0006284-BP-base-excision repair
PF3D7_0917100	GO	GO-0006289-BP-nucleotide-excision repair
PF3D7_0934100	GO	GO-0006289-BP-nucleotide-excision repair
PF3D7_1003700	GO	GO-0006281-BP-DNA repair
PF3D7_1006000	GO	GO-0006284-BP-base-excision repair
PF3D7_1008200	GO	GO-0006281-BP-DNA repair
PF3D7_1011700	GO	GO-0006289-BP-nucleotide-excision repair
PF3D7_1012000	GO	GO-0006281-BP-DNA repair
PF3D7_1037600	GO	GO-0006281-BP-DNA repair
PF3D7_1037600	GO	GO-0006289-BP-nucleotide-excision repair
PF3D7_1106000	GO	GO-0006281-BP-DNA repair
PF3D7_1107400	GO	GO-0006281-BP-DNA repair
PF3D7_1112600	GO	GO-0006281-BP-DNA repair
PF3D7_1117800	GO	GO-0006298-BP-mismatch repair
PF3D7_1129500	GO	GO-0006284-BP-base-excision repair
PF3D7_1140300	GO	GO-0006281-BP-DNA repair
PF3D7_1215700	GO	GO-0006298-BP-mismatch repair
PF3D7_1244200	GO	GO-0006281-BP-DNA repair
PF3D7_1250800	GO	GO-0006289-BP-nucleotide-excision repair
PF3D7_1303800	GO	GO-0006281-BP-DNA repair
PF3D7_1304100	GO	GO-0006281-BP-DNA repair
PF3D7_1314900	GO	GO-0006281-BP-DNA repair
PF3D7_1316900	GO	GO-0006281-BP-DNA repair
PF3D7_1332100	GO	GO-0006281-BP-DNA repair
PF3D7_1332600	GO	GO-0006281-BP-DNA repair
PF3D7_1338400	GO	GO-0006281-BP-DNA repair
PF3D7_1353500	GO	GO-0006281-BP-DNA repair
PF3D7_1368300	GO	GO-0006281-BP-DNA repair
PF3D7_1368800	GO	GO-0006281-BP-DNA repair
PF3D7_1405400	GO	GO-0006298-BP-mismatch repair

PF3D7_1415000	GO	GO-0006284-BP-base-excision repair
PF3D7_1427500	GO	GO-0006298-BP-mismatch repair
PF3D7_1429900	GO	GO-0006281-BP-DNA repair
PF3D7_1430600	GO	GO-0006281-BP-DNA repair
PF3D7_1432600	GO	GO-0006289-BP-nucleotide-excision repair
PF3D7_1441900	GO	GO-0006289-BP-nucleotide-excision repair
PF3D7_1455300	GO	GO-0006298-BP-mismatch repair
PF3D7_1467100	GO	GO-0006284-BP-base-excision repair
PF3D7_1471600	GO	GO-0000724-BP-double-strand break repair via homologous recombination
