# Supplementary material for: K13-Mediated Reduced Susceptibility to Artemisinin in Plasmodium falciparum Is Overlaid on a Trait of Enhanced DNA Damage Repair
Source: Cell Rep. 2020 Aug 4;32(5):107996. doi: 10.1016/j.celrep.2020.107996 (PMC7408483; doi:10.1016/j.celrep.2020.107996)
Supplement: Document S2. Article plus Supplemental Information [file mmc2.pdf]

Cell Reports

Article

K13-Mediated Reduced Susceptibility to Artemisinin in *Plasmodium falciparum* Is Overlaid on a Trait of Enhanced DNA Damage Repair

Graphical Abstract

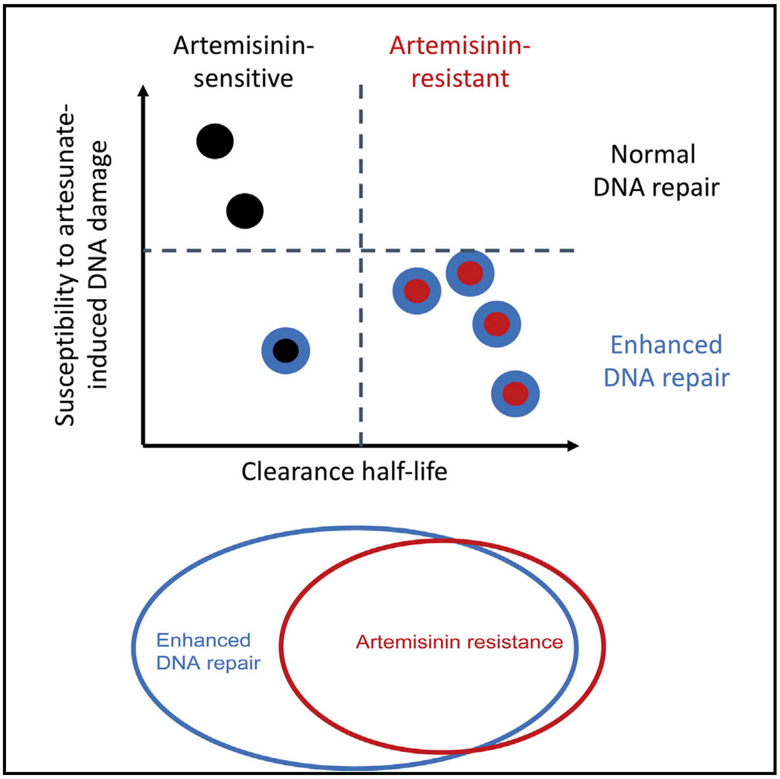

Highlights

- High-throughput MalariaCometChip to measure DNA damage level in *P. falciparum*
- Subpopulation of Cambodian isolates possess enhanced DNA damage repair
- Important link between enhanced DNA damage repair and artemisinin resistance

Authors

Aoli Xiong, Prem Prakash, Xiaohong Gao, ..., Bevin P. Engelward, Jongyoon Han, Peter R. Preiser

Correspondence

prpreiser@ntu.edu.sg

In Brief

Southeast Asia (SEA) has been the breeding ground for malaria drug resistance, including resistance to artemisinin, the first-line anti-malarial drug. In this study, Xiong et al. find a relationship between DNA damage repair and artemisinin resistance in SEA, leading to insights on the establishment and spread of current artemisinin resistance.

Xiong et al., 2020, Cell Reports *32*, 107996
August 4, 2020 © 2020 The Author(s).
https://doi.org/10.1016/j.celrep.2020.107996

CellPress

Cell Reports

CellPress
OPEN ACCESS

Article

K13-Mediated Reduced Susceptibility to Artemisinin in *Plasmodium falciparum* Is Overlaid on a Trait of Enhanced DNA Damage Repair

Aoli Xiong,[1,2,3] Prem Prakash,[1] Xiaohong Gao,[1] Marvin Chew,[1,3] Ian Jun Jie Tay,[5,7] Charles J. Woodrow,[6] Bevin P. Engelward,[5] Jongyoon Han,[2,3,4,5] and Peter R. Preiser[1,3,8,*]

[1]School of Biological Sciences, Nanyang Technological University, 60 Nanyang Dr., Singapore 637551, Singapore
[2]BioSystems and Micromechanics (BioSyM) Interdisciplinary Research Group (IRG), Singapore-MIT Alliance for Research and Technology (SMART), 1 Create Way, Singapore 138602
[3]Antimicrobial Resistance (AMR) IRG, Singapore-MIT Alliance for Research and Technology (SMART), 1 Create Way, Singapore 138602, Singapore
[4]Department of Electrical Engineering and Computer Science, Massachusetts Institute of Technology, 50 Vassar St., Cambridge, MA 02142, USA
[5]Department of Biological Engineering, Massachusetts Institute of Technology, 77 Massachusetts Avenue, Cambridge, MA 02139, USA
[6]Mahidol-Oxford Tropical Medicine Research Unit, Faculty of Tropical Medicine, Mahidol University, 420/6 Rajvithi Road, Tungphyathai, Bangkok 10400, Thailand
[7]Present address: Institute of Molecular and Cellular Biology, ASTAR, Singapore, Singapore
[8]Lead Contact
*Correspondence: prpreiser@ntu.edu.sg
https://doi.org/10.1016/j.celrep.2020.107996

SUMMARY

Southeast Asia has been the hotbed for the development of drug-resistant malaria parasites, including those with resistance to artemisinin combination therapy. While mutations in the kelch propeller domain (K13 mutations) are associated with artemisinin resistance, a range of evidence suggests that other factors are critical for the establishment and subsequent transmission of resistance in the field. Here, we perform a quantitative analysis of DNA damage and repair in the malaria parasite *Plasmodium falciparum* and find a strong link between enhanced DNA damage repair and artemisinin resistance. This experimental observation is further supported when variations in seven known DNA repair genes are found in resistant parasites, with six of these mutations being associated with K13 mutations. Our data provide important insights on confounding factors that are important for the establishment and spread of artemisinin resistance and may explain why resistance has not yet arisen in Africa.

INTRODUCTION

Malaria remains a severe public health burden, causing an estimated 228 million cases and about 0.4 million deaths in 2018 (World Health Organization, [WHO], 2019). Currently, artemisinin-based combination therapies (ACTs) are the first-line antimalarial treatment recommended by WHO (2019). However, parasites resistant to artemisinin have emerged in Southeast Asia (SEA), leading to significant selection pressure on the partner drugs used in ACTs (Amaratunga et al., 2014). There is now evidence that resistance to piperaquine, the partner drug used in Cambodia, is indeed developing (Amaratunga et al., 2016; Chaorattanakawee et al., 2015; Duru et al., 2015; Leang et al., 2015; Witkowski et al., 2017). Phenotypically, resistant parasites exhibit an increased clearance half-life in patients and higher survival rates in the *in vitro/ex vivo* ring-stage survival assay (RSA) (Amaratunga et al., 2014; Witkowski et al., 2013). Mutations in the kelch propeller domain (K13 mutations) are associated with artemisinin resistance in SEA (Ariey et al., 2014; Straimer et al., 2015) but not yet in Africa. While polymorphisms in the K13 gene are found in Africa, they have not led to resistance so far (Kamau et al., 2015; Taylor et al., 2015). However, there have been case reports on infected travelers returning from Africa responding poorly to ACTs, with some carrying resistance-associated K13 mutations, indicating an early sign of resistance development (Russo et al., 2018; Sondén et al., 2017; Sutherland et al., 2017; Lu et al., 2017; Rasmussen et al., 2017; Van Hong et al., 2014). Indeed, evidence now shows >5% prevalence of artemisinin resistance associated K13 mutations in Rwanda, Guyana, and Papua New Guinea, but the clearance and efficacy of first-line treatment in the regions have not yet been affected (WHO, 2019).

From the available data, it is clear that a single SNP in K13 cannot be solely responsible for the establishment and spread of artemisinin resistance in the field (AlKadi, 2007; Gil et al., 2003). Rather, it suggests a complex trait prevalent in SEA, with mutations in ferredoxin (fd), multidrug resistance protein 2 (mdr2), chloroquine resistance transporter (crt), and apicoplast

This is an open access article under the CC BY license (http://creativecommons.org/licenses/by/4.0/).

ribosomal protein S10 (arps10), being linked with K13 mutations and resistance (Miotto et al., 2015). In fact, SEA has long been the hotbed for drug resistance development (Dondorp et al., 2010). In addition to high drug pressure caused by extensive drug use and widespread counterfeit drugs in the area, earlier research also attributed this to the hypermutability or accelerated resistance to multiple drugs (ARMD) phenotype, whereby the parasite exhibited 1,000-fold higher rates of resistance to anti-malarial drugs compared with wild-type (WT) strains (Castellini et al., 2011). The phenotype was primarily linked to defects in DNA repair; most likely, DNA damage sensing (Gupta et al., 2016). Although the debate on the relationship between the ARMD phenotype and drug resistance development in SEA has continued, altered DNA repair has been linked to the resistance development in SEA (Castellini et al., 2011; Lee and Fidock, 2016).

As there are no equivalent alternatives to ACTs approved for clinical use, it is critical that we obtain a better understanding of all the factors that contribute to artemisinin resistance to facilitate better surveillance, containment, and prevention of artemisinin resistance. It has been shown that artesunate (a derivative of artemisinin) can induce DNA damage in trophozoite-stage malaria parasites after only 1 h of treatment and that DNA repair pathways are activated and able to repair damage caused by sub-lethal doses of artesunate (Gopalakrishnan and Kumar, 2015; Gupta et al., 2016).To explore the role of DNA damage repair in artemisinin resistance in the field, we adapted the previously developed CometChip, a high-throughput platform based on the well-established comet assay (Singh et al., 1988; Wood et al., 2010; Ge et al., 2012, 2014, 2015; Sykora et al., 2018; Weingeist et al., 2013), for the quantitative assessment of DNA damage in *P. falciparum* and analyzed lab-generated K13-resistant parasites and culture-adapted Cambodian clinical isolates from the Tracking Resistance to Artemisinin Collaboration, collected in Pailin, Cambodia, in 2011 (Amaratunga et al., 2014; Ashley et al., 2014). We further surveyed the available genome sequence data and identified a number of mutations in known DNA damage repair genes that are associated with the K13-resistant phenotype. Overall, we provided important insights on the important role of altered DNA repair in the establishment of artemisinin resistance in SEA. The information not only gives us better understanding of current artemisinin resistance but will also be valuable for surveillance as well as containment of artemisinin resistance.

RESULTS

Detection of Artesunate-Induced DNA Damage in Early and Late Stage *P. falciparum* Using Alkaline malariaCometChip

Previous work has suggested that artesunate induces DNA damage in the *P. falciparum* parasite via the formation of reactive oxygen species (ROS) (Gopalakrishnan and Kumar, 2015). Another study also showed that direct damage on purified plasmid DNA can be caused by a high concentration (non-physiologically relevant) of activated artemisinin *ex vivo* (Wu et al., 1996). To obtain a more comprehensive understanding of artesunate-induced DNA damage, we first investigated the effect of artesunate, activated with ferrous heme or non-heme exogenous Fe^{2+}, on purified DNA using more physiologically relevant concentrations. Our results showed that a lower concentration of activated artesunate did not induce single-strand breaks (SSBs) or double-strand breaks (DSBs) on purified DNA as compared with hydrogen peroxide (Figure S1), which suggests that the effect of artesunate on cellular DNA inside the parasite involves further escalating of artesunate-relevant radicals and a (so far) poorly understood radical-related damaging mechanism. Since previous studies on artesunate-induced DNA damage used the classic comet assay (Gopalakrishnan and Kumar, 2015), this suggested that the damage could also be detected and assessed more easily using CometChip, a high-throughput platform previously developed for the study of DNA damage in mammalian cells based on the principle of the comet assay (Wood et al., 2010; Ge et al., 2012, 2014, 2015; Sykora et al., 2018; Weingeist et al., 2013).

To adapt and optimize the CometChip platform for the study of malaria parasites, we first utilized hydrogen peroxide to induce DNA damage in the *P. falciparum* clone 3D7. In principle, as the amount of DNA damage increases (e.g., SSB, abasic site, etc.), DNA migration in electrophoresis under alkaline conditions increases (Singh et al., 1988). Therefore, the percentage of DNA in the "comet" tail (% tail DNA), quantified as described in the Supplemental Information and Figure S2, was used to represent the level of DNA damage. Due to the relatively small genome size of malaria parasites, it is necessary to concentrate the parasites in the infected blood samples to obtain enough total DNA for reliable detection. For this, ring-stage parasites (0–18 h post-infection; h.p.i.) were treated with streptolysin O (SLO) as previously described (Jackson et al., 2007; Külzer et al., 2015) to obtain a highly enriched ring stage culture. These ring-stage parasites were loaded onto the CometChip, sealed with low-melting-point agarose (LMPA), and treated for 20 min with increasing concentrations of hydrogen peroxide followed by immediate alkaline lysis. An observable "comet" was already detected at the lowest (100 μM) hydrogen peroxide concentration and increased further as the concentration of hydrogen peroxide was raised (Figure 1A). The % tail DNA was quantified, and the results demonstrated a dose-dependent increase of % tail DNA in response to hydrogen peroxide in ring-stage parasites (Figure 1C). To study DNA damage in more mature parasites, schizont-stage parasites (estimated 30–48 h.p.i.) were concentrated using Percoll gradient centrifugation before ComentChip loading and hydrogen peroxide treatment. Similar to what had been observed with ring-stage parasites, hydrogen peroxide concentration-dependent increase of the % tail DNA was observed (Figures 1B and 1C). The data obtained with hydrogen peroxide suggested successful adaptation of the CometChip platform (malariaCometChip) in investigating the level of DNA damage in *P. falciparum* parasites.

We then utilized the malariaCometChip to investigate the impact of artesunate on ring- and schizont-stage parasites. Enriched ring- and schizont-stage *P. falciparum* 3D7, *P. falciparum* laboratory strains Dd2 (denoted as "Dd2"), and K13 I543T mutant (derived from Dd2, denoted as "$Dd2^{I543T}$") were investigated. Ring- and schizont-stage parasites were treated with different concentrations of artesunate, respectively, for 1 h

CellPress
OPEN ACCESS

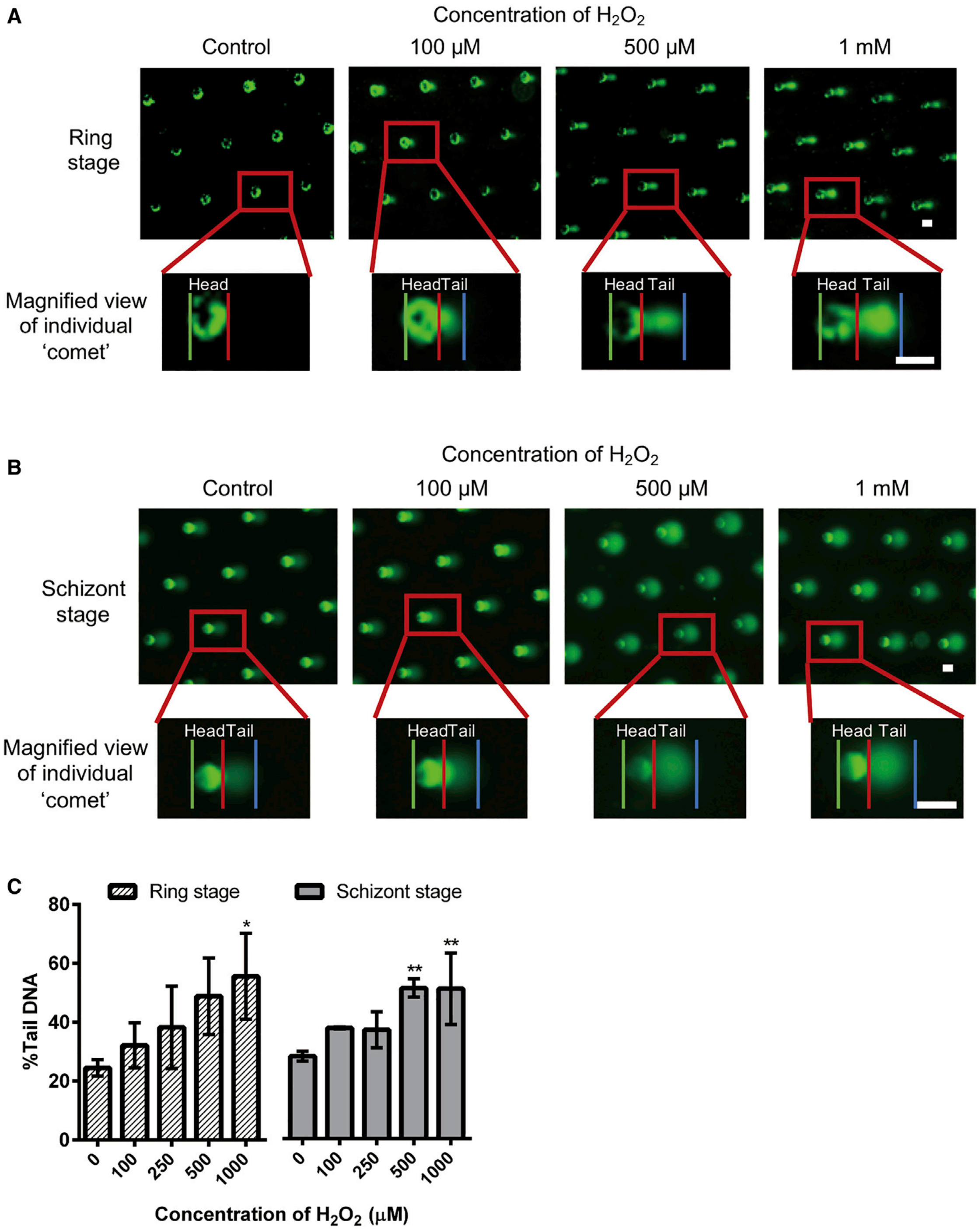

Figure 1. Optimization of CometChip

(A and B) Representative pictures of "comet" of ring and schizont-stage *P. falciparum* 3D7 after on-chip hydrogen peroxide (H_2O_2) treatment. Each green dot indicates a microwell in the CometChip. Each macrowell of the 96-well plate contains around ~300 of such microwells. The magnified view of the boxed area below shows the differentiation of head (between the green line and red line) and tail (between the red line and blue line) of an individual comet. This process would be automatically done by the algorithm as shown in the Supplemental Information. Scale bars, 50 nm.

(C) Quantification of "comet" after treatment. The y axis represents the percentage of DNA at the "comet" tail (%Tail DNA). On average, ~306 comets were analyzed per macrowell. Each experiment was done in technical triplicates, and data here were collected and are shown in biological triplicates. $^{*}p < 0.05$; $^{**}p < 0.01$, one-way analysis of variance (ANOVA). Dunnett's multiple comparison tests were performed between vehicle control (0 µM) and respective concentrations. All data are indicated as means ± SEM.

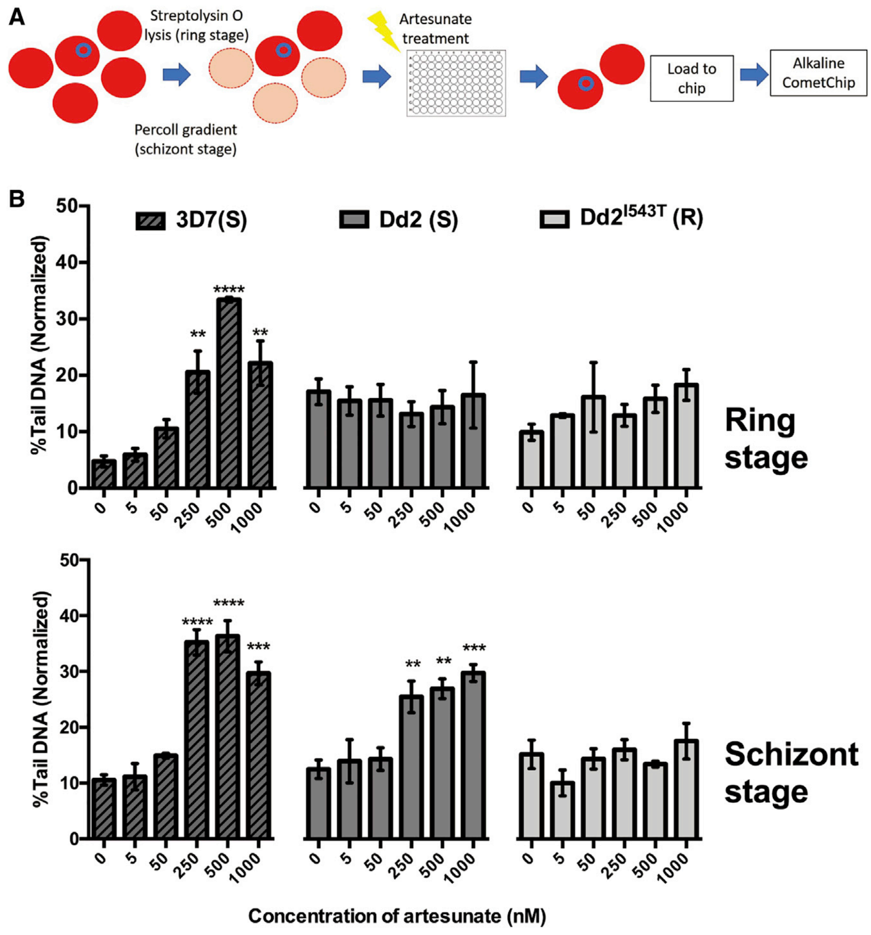

Figure 2. Artesunate-Induced DNA Damage in *P. falciparum* Lab Strains

(A) Schematic illustration of artesunate treatment based on Streptolysin O (SLO) enrichment (ring stage) or Percoll gradient centrifugation (schizont stage).

(B) Artesunate-induced DNA damage in *P. falciparum* 3D7, Dd2, and Dd2^{I543T}. Ring-stage (upper panel) and schizont-stage (lower panel) *P. falciparum* parasites were treated with different concentrations of artesunate for 1 h and subjected to analysis on alkaline CometChip. On average, ~302 comets were analyzed per macrowell. Each experiment was done in technical triplicates, and data here were collected and are shown in biological triplicates or quadruplicates. **p < 0.01; ***p < 0.001; ****p < 0.0001, one-way ANOVA. Dunnett's multiple comparison tests were performed between vehicle control (0 μM) and respective concentrations. All data are indicated as means ± SEM.

in vitro before transfer onto the CometChip analkaline lysis (ring stage: 0–18 h.p.i.; schizont stage: 30–48 h.p.i.; Figure 2A). The time gap between artesunate treatment and on-chip alkaline lysis was limited to 5 min.

Clear concentration-dependent DNA damage was observed in both ring- and schizont-stage 3D7 parasites (Figure 2B), demonstrating that artesunate-induced DNA damage can be detected and quantified on the malariaCometChip. Unlike in the case of the 3D7 strain, no artesunate-induced DNA damage can be observed in ring-stage Dd2 and Dd2^{I543T} (Figure 2B). In contrast, schizont-stage Dd2 exhibited a dose-dependent increase of the DNA damage, while Dd2^{I543T} showed a significantly lower amount of DNA damage (Figure 2B). The difference between the 3D7 and Dd2 strains and the difference between schizont-stage resistant and sensitive Dd2 strains indicate that an increased ability of parasites to deal with artesunate-induced DNA damage may enhance the survival chances of parasites and thus play an important role in resistance establishment.

DNA-Damage-Resistant Phenotype and Field Artemisinin Resistance

To explore this further, 7 culture-adapted clinical isolates from Pailin, Cambodia, were tested for artesunate-induced DNA damage at the schizont stage (Figures 3A and 3B). Resistant isolates KH004-003 (KH03), KH004-032 (KH32), and KH004-048 (KH48) showed no significant changes in DNA damage even at a 1,000-nM drug concentration, while the resistant strain KH004-014 (KH14) even showed a reduction in DNA damage at a higher concentration of artesunate treatment (Figure 3A). Sensitive isolates KH004-041 (KH41) and KH004-042 (KH42) showed significantly higher DNA damage at a 1,000-nM drug concentration, while surprisingly, the sensitive strain KH004-044 (KH44) showed a reduction or no significant change in DNA damage after artesunate treatment. The level of DNA damage at 1,000 nM artesunate treatment in different KH strains was found to be inversely correlated with their corresponding reported clinical clearance half-lives (Mok et al., 2015) (Figure 3C). Based on clinical criteria, the resistant parasites are separated from sensitive ones at a clearance half-life >5 h. With 21.7% normalized tail DNA as the separation criterion on the y axis, four zones were created and labeled counterclockwise as I, II, III, and IV. With zone II representing false-negative and zone IV representing false-positive results, 6 out of 7 strains tested located to the expected zones I and III, with the exception of KH44, which was located in zone II, indicating that resistant parasites as well as some sensitive parasites have an ability to deal more effectively with DNA damage induced by artesunate.

To investigate whether this resistance to DNA damage is artesunate specific or represents a broader adaptation of the parasite to deal with DNA-damage-inducing agents, we treated KH44 and KH03 as well as 3D7 and KH41 with methyl methane sulfonate (MMS), which is known to methylate DNA bases and stall the replication forks, causing SSBs and DSBs (Lundin et al., 2005). After treatment as short as 30 min, clear MMS-induced DNA damage was observed in 3D7 and the sensitive KH41, but not in KH44 and the resistant KH03 parasites

CellPress
OPEN ACCESS

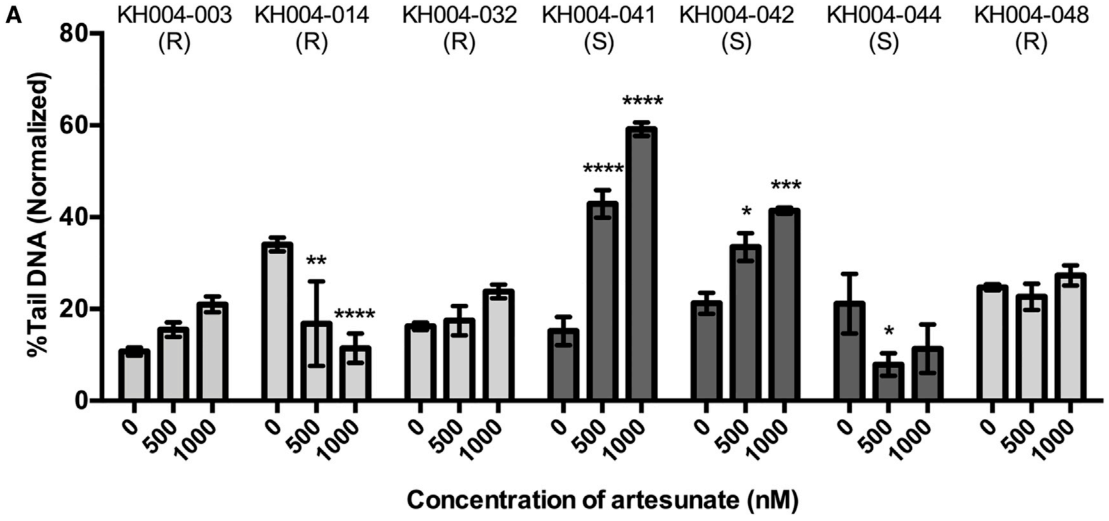

B

	KH004-003	KH004-014	KH004-032	KH004-041	KH004-042	KH004-044	KH004-048
K13 (KP/BTB)	2	2	2	1	1	1	2
crt-N326S	N/A	2	2	0	1	2	2
crt-I356T	N/A	2	2	1	1	2	2
fd-D193Y	N/A	2	2	1	1	2	2
mdr2-T484I	N/A	2	2	1	3	2	2
Clearance half-life (h)	8.33	8.26	7.91	1.61	2.8	3.18	6.9

Note: 0=missing (i.e. less than 5 sequencing reads at that position); 1=reference allele (same as 3D7); 2=mutant allele (column 1 for resulting mutation); N/A=not applicable (i.e. data not reported or collected)

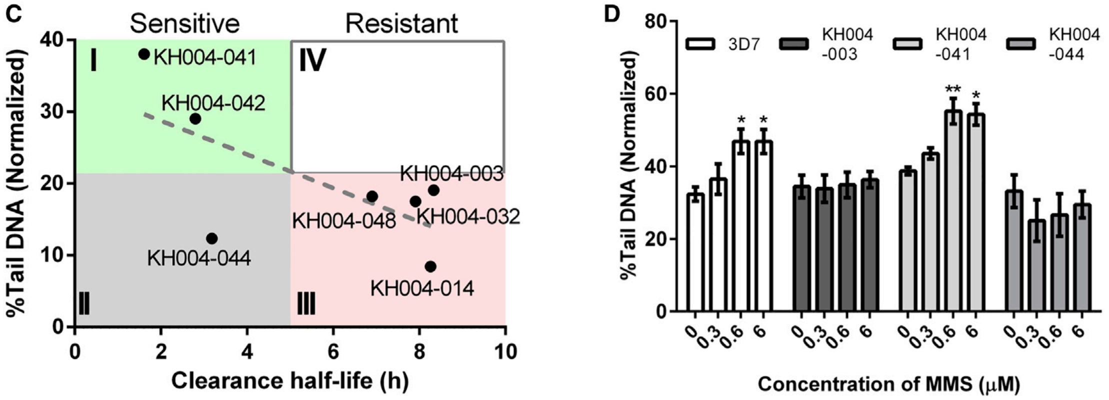

Figure 3. DNA Damage Resistance Phenotype in Cambodian Clinical Isolates

(A) Artesunate-induced DNA damage in Cambodian KH004 clinical isolates. Schizont-stage KH isolates were treated with different concentrations of artesunate for 1 h. R represents artemisinin-resistant isolates, and S represents sensitive isolates. On average, ~316 comets were analyzed per macrowell. Each experiment was done in technical triplicates, and data here were collected and are shown in biological triplicates. *$p < 0.05$; **$p < 0.01$; ***$p < 0.001$, two-way ANOVA. Comparisons were made between vehicle control and each concentration, and p values were adjusted after Šidák correction.

(B) Information of the KH isolates used in this work. Information on mutations and clearance half-lives was provided by TRAC or extracted from its related study (Mok et al., 2015). N/A, not applicable (e.g., data were not reported).

(C) Correlation between DNA damage level at 1,000 nM artesunate and parasite clearance half-life in multiple clinical isolates. Linear regression was performed using Prism, and the dotted line indicates the linear fit curve. 5-hour clearance half-life is defined as the cutoff between artemisinin-resistant and sensitive parasites. The intersect between 5-hour clearance half-life and the linear fit curve is at %Tail DNA equal to 21.7%. Four zones were thus divided by 5 h of clearance half-life and 21.7% tail DNA and are labeled anti-clockwise as I, II, III, IV from upper left corner.

(D) Methyl methane sulfonate (MMS)-induced DNA damage. Schizont-stage parasites *P. falciparum* 3D7, artemisinin-resistant KH004-003, and artemisinin-sensitive KH004-041 and KH004-044 were treated with different concentrations of MMS for 30 min. ~411 comets were analyzed per macrowell. Each experiment was done in technical triplicates, and data here were collected and are shown in biological triplicates. *$p < 0.05$; **$p < 0.01$, two-way ANOVA. Comparisons were made between vehicle control and each concentration, and p values were adjusted after Šidák correction. All data are indicated as means ± SEM.

(Figure 3D), suggesting that the latter has greater intrinsic ability to deal with DNA damage instead of specifically dealing with an artesunate-induced one.

Taken together, our results suggest that some Cambodian field isolates are able to deal more effectively with DNA damage, possibly through a better repair mechanism. This ability to resist DNA damage is seen in all of the artemisinin-resistant parasites as well as a fraction of sensitive parasites. In other words, a subset of DNA-damage-resistant parasites may have developed artemisinin resistance, raising the question on whether the ability to better adapt to DNA damage is an important factor in developing resistance to artesunate.

Candidate Molecular Markers for Altered DNA Repair Capability

Previous work suggested that parasites defective in DNA damage repair, where repair accuracy is compromised due to increased repair speed, may contribute to higher levels of mutations and, by proxy, an increased rate in the development of drug resistance in SEA (Miotto et al., 2013; Castellini et al., 2011; Lee and Fidock, 2016). To understand the underlying genetic factors contributing to the lower level of DNA damage observed in KH44 and the other resistant parasites as compared with sensitive strains, we constructed a phylogenetic tree grouping samples from Pailin, Cambodia, 2011, including the KH samples used in this study, according to their genome-wide SNP similarity using available SNP data from the Pf3K database (Ashley et al., 2014; Miotto et al., 2015) (Figure S4). Among the KH strains, KH41, KH42, and KH44 have WT *kelch13* and are considered artemisinin sensitive. KH41 and KH42, which showed DNA damage response to artesunate (Figure 3A), were closely related to each other and only distantly related to the resistant KH strains. However, KH44, which showed no DNA damage at high concentrations of artesunate treatment, clustered with resistant strains and was most closely related to the resistant strain KH14, which showed a similar decrease of comet tails upon artesunate treatment (Figure 3A).

To investigate further, mutations in known DNA repair genes (Table S3) were compared in these clinical isolates. Considering KH44 as repair enhanced due to the low level of DNA damage it experienced after artesunate treatment (Figure 3A), seven mutations in six genes were found to be different between the five DNA damage repair enhanced KH strains and sensitive KH41 and KH42 (Table 1; Table S1), including PF3D7_0710400 encoding DNA repair protein RAD14, which plays an important role in nucleotide excision repair and DNA damage recognition; PF3D7_100600 for IMP1-like protein involved in base excision repair; PF3D7_1106000 for RuvB-like helicase 2, which has ATP-dependent 5′-3′ DNA helicase activity; PF3D7_1368800 for DNA repair endonuclease XPF; PF3D7_1429900 for ADP-dependent DNA helicase RecQ; and PF3D7_1455300 for a conserved Plasmodium protein that plays a role in DNA mismatch repair. Among the seven genes, three of them—PF3D7_0710400, PF3D7_1106000, and PF3D7_1368800—were classified as essential according to previous work (Zhang et al., 2018), and PF3D7_1106000 was previously linked to resistance development (Miotto et al., 2013, Lee and Fidock, 2016).

For the subsequent analysis presented here, seven mutations in six genes were defined as "EnDNArep genotype," and their counterparts in WT K13 carrying parasites as "NormalDNArep genotype." Notably, WT Dd2, the ARMD strain that showed a lower level of DNA damage compared with KH41 at both the ring and schizont stages, possess three of the seven mutations in the EnDNArep genotype (Table S1), suggesting a potential link between the EnDNArep genotype and ARMD phenotype.

EnDNArep Genotype and Development of Field Artemisinin Resistance

To investigate whether the EnDNArep genotype is linked to K13 mutations and artemisinin resistance, linkage disequilibrium (LD) was assessed between K13 and each EnDNArep mutation using chi-square analysis, and the p values were plotted (Figure 5A). Heterozygotes (due to mixed infection) were excluded from the analysis. After Bonferroni correction on the p value, five out of seven mutations were found to be associated with K13 mutations in the SEA population with statistical significance, except for SNPs at PF3D7_0710400-474979 and PF3D7_1455300. Previous studies suggested a strong regional difference between parasite populations from the eastern and western Greater Mekong Sub-regions (e-GMS and w-GMS, respectively) in SEA (Miotto et al., 2013, 2015). LD was thus assessed between the EnDNArep genotype and K13 mutations after further dividing the SEA population into e-GMS and w-GMS groups, respectively (Figure 4). Consistent with previous observations, differences in gene associations were observed between the two regions. In e-GMS, where the KH strains analyzed in this study come from, six out of seven mutations were found to be highly associated with K13 mutations, except for SNPs at PF3D7_1455300 (Figure 5A). In contrast, only three out of seven mutations, including the SNPs at PF3D7_1106000 and the deletions in PF3D7_1006000 and PF3D7_1429900, were found to be associated with K13 mutations in w-GMS (Figures 5A and 5C).

The distribution of the EnDNArep genotype in clinical isolates from different areas with different K13 mutation prevalence was analyzed. With the exception of SNPs at PF3D7_0710400-475135 and PF3D7_1455300, the frequency of the other mutations in the EnDNArep genotype decreased along with the frequency of K13 mutations across the areas in e-GMS (Figure 5B). In Pailin, where around 90% of the parasites carry K13 mutations—mostly C580Y—a high proportion of EnDNArep genotype was observed in the K13 parasites (Figure 4). In study sites with lower K13 mutation prevalence, the percentage of K13 parasites having EnDNArep genotype also becomes lower. For example, in Pursat, Cambodia, which has a lower prevalence of K13 mutations (77.32%) compared with Pailin, more than 60% of the K13 parasites have the mutations in the EnDNArep genotype except for PF3D7_1368800 (41.67%) (Figure 5). In Phuoc Long, where half of the population carry K13 mutations, only four out of seven mutations in the EnDNArep genotype were found to be over 50% in the K13 population (Figure 5). From a temporal perspective, most of the resistant parasites from Pailin, 2011, including the four KH-resistant strains investigated here, shared a similar EnDNArep genotype (average, around 80%), especially for PF3D7_1006000 (95.65%) and PF3D7_1455300 (100%) (Figure S5). In 2012, as the frequency of K13 mutations

CellPress
OPEN ACCESS

Table 1. List of Mutations Identified Different between DNA-Damage-Resistant and -Sensitive Isolates

Gene ID	Position	Reference	Variant	Type	Variant Type	Impact	Amino Acid Change	Product	Predict Function	Gene Essentiality
PF3D7_ 0710400	474979	TA	*	DEL	missense_ variant	moderate	–	DNA repair protein RAD14, putative	nucleotide excision repair, DNA damage recognition	high
	475135	A	G	SNP	missense_ variant	moderate	Ile171 Met			
PF3D7_ 1006000	257175	TAATCTT TCCCTTT CTTTTTC TTCTTCC AATAAT TTC	T	DEL	frameshift_ variant& missense_ variant	high	Lys1 64fs	IMP1-like protein, putative	unknown function	low
PF3D7_ 1106000	257027	T	C	SNP	missense_ variant	moderate	Asn39 Asp	RuvB-like helicase 2	ATP-dependent 5′-3′ DNA helicase activity	high
PF3D7_ 1368800	2731444	TCGGTG GTCCAT TTTTTT CAACTT CCAGGT GGTCA TTTGTTT GAACTT CCAGAT GGTT	*	DEL	frameshift_ variant& missense_ variant	high	Glu 1567fs	DNA repair endonuclease XPF, putative	DNA repair, nuclease activity	high
PF3D7_ 1429900	1178944	TGATAAAG	T,*,TGATAATG	DEL	frameshift_ variant& missense_ variant	high	Asn 725fs	ADP-dependent DNA helicase RecQ	ATP-dependent 3′-5′ DNA helicase activity	low
PF3D7_ 1455300	2260945	T	G	SNP	missense_ variant	moderate	Met4 94Arg	conserved Plasmodium protein	mismatch repair, single-stranded DNA 5′-3′ exodeoxyri bonuclease activity, flap endonuclease activity	low

Mutation type, variant type, impact, and amino acid change were predicted using SnpEff (Cingolani et al., 2012) with the latest *P. falciparum* 3D7 genome data from PlasmoDB. Information on gene product, predict function, and gene essentiality (Zhang et al., 2018) was extracted from PlasmoDB. Asterisks (*) represent deletion of the referene DNA.

remained high and the K13 C580Y mutation dominated in the area, the K13 parasites from Pailin further progressed, with an increased percentage of resistant parasites possessing the EnDNArep genotype (average, around 90%) (Figure 5), indicating an interplay between K13 mutations, especially C580Y, and the EnDNArep genotype in the K13-mediated artemisinin resistance.

The distribution of the EnDNArep genotype in WT parasites showed a broad similarity between areas in SEA but is clearly different in Africa. In places like Pailin and Pursat, where artemisinin resistance is prevalent, more than half of the WT parasites were found to possess the majority of the EnDNArep genotype (Figure 5). In Ramu, Bangledash, while geographically linked to Pailin but with no K13 mutation documented in 2012, four out of the seven mutations in the EnDNArep genotype were found in more than half of the WT parasites. In contrast, in the African samples, where only one K13 mutation was found in 111 cases representing Africa (mainly from Ilorin, Nigeria, and Kinshasa, Democratic Republic of Congo) (Ashley et al., 2014) and with clear geographic separation from SEA, only one out of the seven mutations in the EnDNArep genotype were found in more than half of the WT population.

A recent study showed that the K13 C580Y mutation gradually dominated other K13 mutations in the resistant population and is likely linked to higher fitness or transmission of the C580Y strains (Imwong et al., 2017). Temporal analysis in Pailin also showed an increased proportion of the EnDNArep genotype in the K13 population when C580Y became the only K13 mutation in the Pailin population from 2011 to 2012 (Figure S5). To investigate whether the EnDNArep genotype is associated with C580Y, the C580Y carrying parasites in the two sub-populations were examined. The results showed a higher percentage of parasites carrying the EnDNArep genotype in the C580Y parasites, especially in

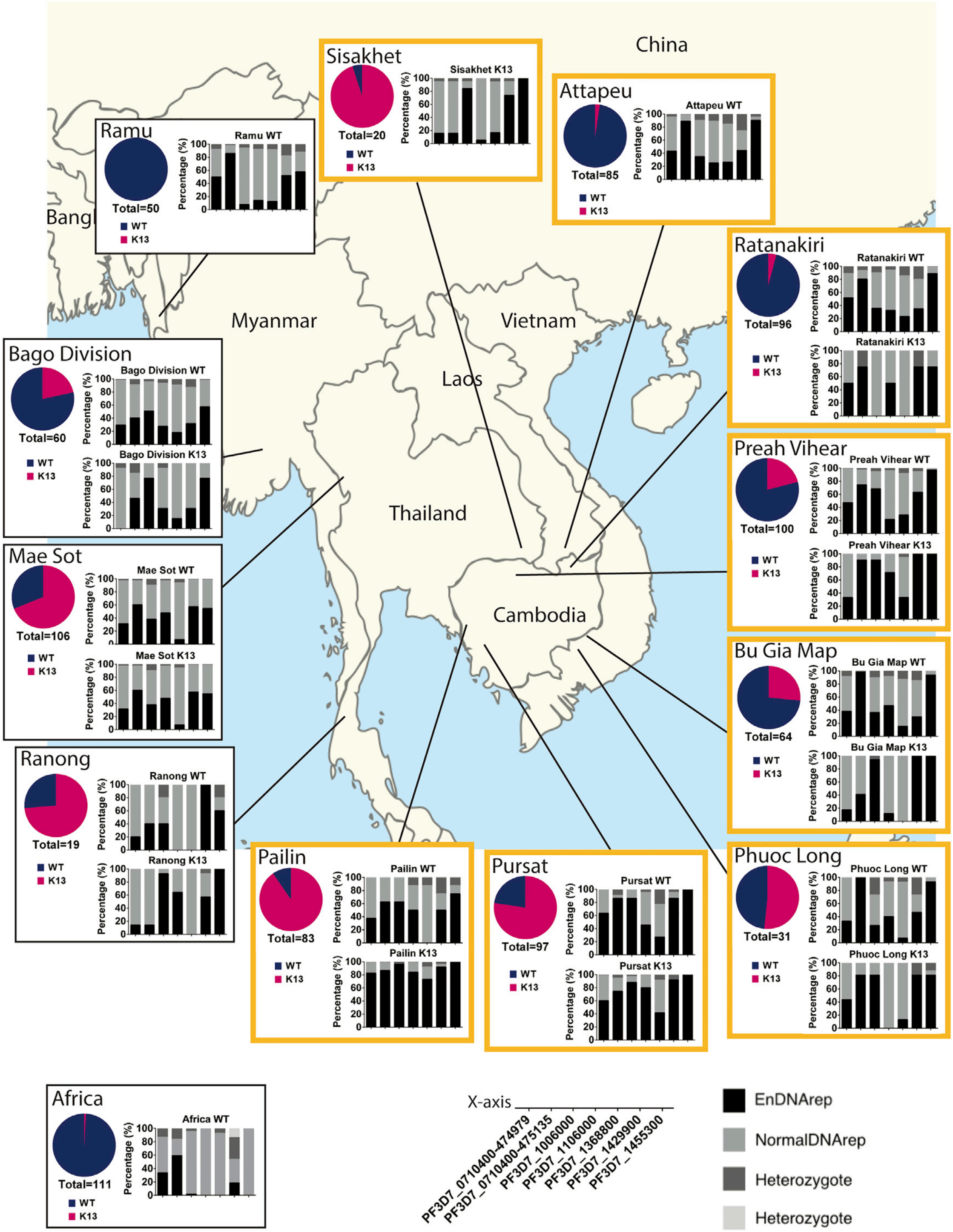

Figure 4. Distribution of the EnDNArep Genotype in SEA and Africa

Area profile of K13 mutation prevalence (pie chart of WT, indicated in blue, and K13, indicated in magenta) and distribution of the EnDNArep genotype in parasites carrying WT K13 (WT; upper panel) and parasites carrying K13 mutations (K13; lower panel) in SEA and Africa. The x axis key and the bar chart key appear at the bottom of the figure. Areas belonging to e-GMS are indicated in orange squares. 111 cases representing Africa were mainly from Ilorin, Nigeria, and Kinshasa, Democratic Republic of Congo (Ashley et al., 2014).

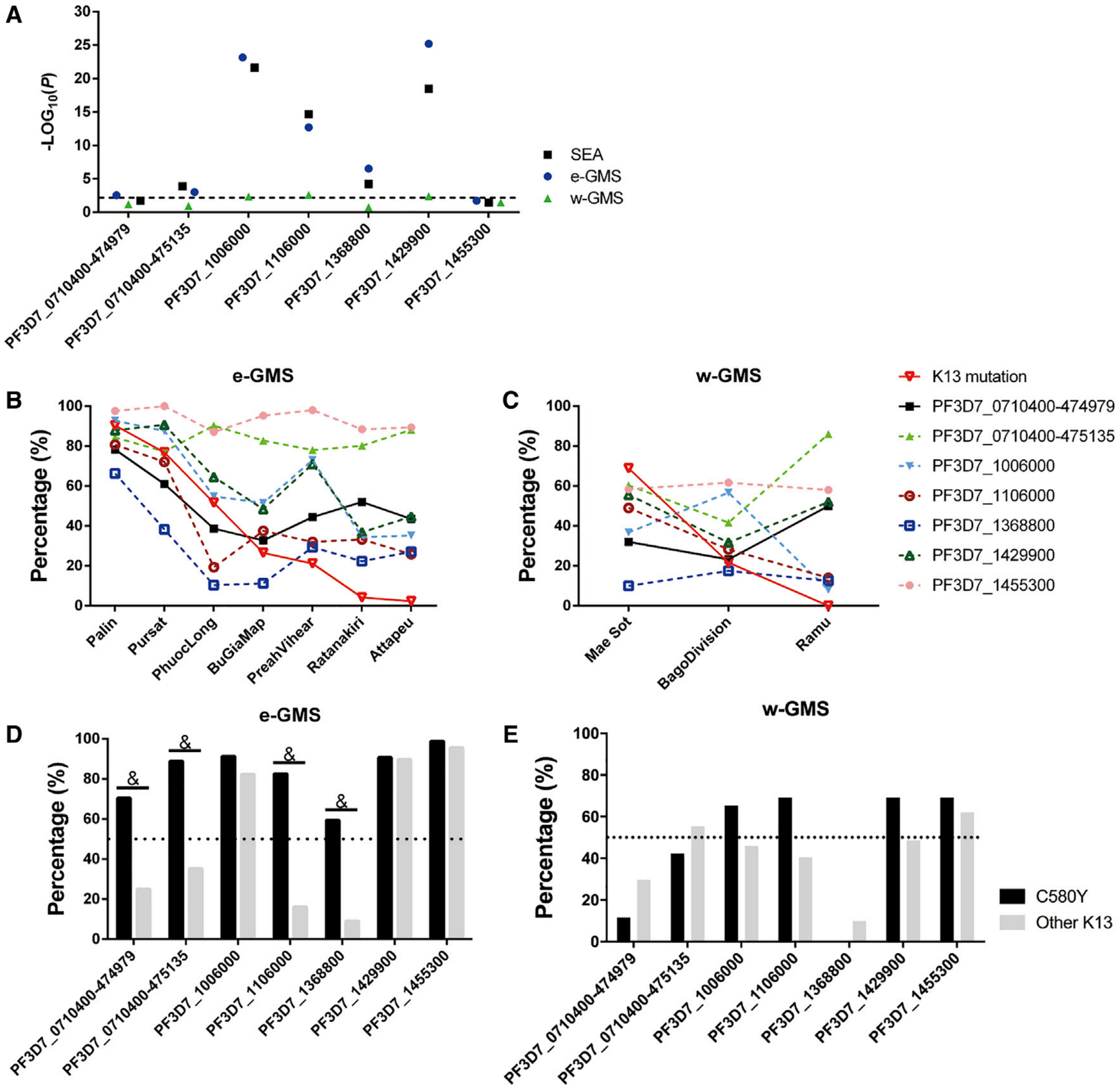

Figure 5. Association of the EnDNArep Genotype with K13 Mutations in SEA

(A) Chi-square analysis of gene association in different populations and sub-populations. SEA refers to the Southeast Asia population, which was further divided into e-GMS and w-GMS. The line was drawn at $p = 0.00714$, the y axis threshold after Bonferroni correction. Heterozygotes due to mixed infection were excluded from analysis.

(B and C) Percentage of the EnDNArep genotype in the population from e-GMS (B) and w-GMS (C) along with the frequency of K13 mutations. Sisakhet and Ranong were excluded due to limited sample size (20 and 19 samples, respectively).

(D and E) Distribution of the EnDNArep genotype in K13 C580Y parasites and parasites with other K13 mutations in e-GMS (D) and w-GMS (E). $^{\&}p < 0.00714$ (value after Bonferroni correction), Chi-square analysis between C580Y and other K13 mutations within the K13 population. The dotted line was drawn at 50%.

e-GMS (Figures 5D and 5E). Analysis of LD between C580Y and other K13 mutations showed a close correlation between K13 C580Y mutation and the SNPs at PF3D7_0710400-475135 and PF3D7_1106000 and the deletions at PF3D7_0710400-474979 and PF3D7_1368800 in e-GMS (Figure 5D). In w-GMS, no significant association can be seen (Figure 5E).

Taken together, the data obtained here show a strong association between enhanced DNA repair and K13-mediated artemisinin resistance in SEA, especially e-GMS. Despite other factors, such as transmission pattern, human migration, and local health policies that may have contributed to such association, the EnDNArep genotype along with previously identified mutations (Miotto et al., 2015), may be part of the predisposing genetic background that is important for the initial establishment and subsequent spread of K13-mediated artemisinin resistance.

DISCUSSION

In this study, we investigated the ability of both lab-adapted parasites and clinical isolates to deal with artesunate-induced DNA damage using the optimized malariaCometChip (Wood et al., 2010). The experimental data showing differences in DNA repair

suggested that there are differences in the genetic background of these parasites that may provide them with an advantage for the development of artemisinin resistance in SEA. Here, we identified seven mutations, including nonsynonymous SNPs and deletions, in known DNA damage repair genes that may contribute to provide the right genetic background for the development of artemisinin resistance in SEA.

Our study has several important observations and implications. First, we observed a significantly improved ability of the schizont-stage, artemisinin-resistant parasite to deal with DNA damage due to short artesunate exposure. While the established phenotype of reduced artemisinin susceptibility is defined only in early ring-stage intra-erythrocytic parasites (Burrows, 2015, Dondorp et al., 2009, Tilley et al., 2016, Witkowski et al., 2013), the improved ability of the resistant parasite to overcome DNA damage suggests an overall better ability to deal with artesunate-induced damage, regardless of stages. This may be important, as without an enhanced DNA repair, K13 mutant parasites that can survive exposure to artemisinin and sustained DNA damage at the ring stage could still fail to mature or produce viable progeny merozoites and thus be less likely to thrive. Therefore, enhanced DNA repair is more likely to be a fitness-enhancing trait for the resistant parasite. Second and most importantly, the enhanced DNA repair was also observed in the unique artemisinin-sensitive clinical isolate KH44, which, similar to resistant isolates, suffered no DNA damage from both artesunate and MMS treatment (Figures 3A and 3C). Of note, KH44 possesses SNP in *fd*, *mdr2*, and *crt*, the markers of the genetic background for contemporary artemisinin resistance development (Miotto et al., 2015). The existence of KH44 suggests that enhanced DNA repair could be a predisposing background for resistance to establish and subsequently spread in the field rather than something that developed afterward.

Analysis of KH44 and resistant isolates allowed us to identify seven mutations in known DNA repair genes that are linked to K13 mutations in the field. Except for the SNP in PF3D7_1106000, other mutations identified here have not been reported to be associated with artemisinin resistance. Top-ranked LD with K13 mutations was found in deletions in PF3D7_1006000 (IMP1-like protein) and PF3D7_1429900 (ADP-dependent DNA helicase RecQ) in both e- and w-GMS. Protein coded by PF3D7_1006000 was found to have mRNA binding activity at the trophozoite and schizont stages without information on protein structure. The deletion in PF3D7_1429900 is predicted to be in the intrinsically disordered region (IDR) of the protein. IDRs are thought to serve as flexible platforms for protein-protein interaction (Strzyz, 2018), indicating a possible impact of the deletion on the protein function and the pathway involved. While these genes are predicted to be non-essential and have a high mutant fitness score *in vitro* (Zhang et al., 2018), this does not preclude them from providing essential functions under oxidative stress or at other stages of the life cycle. Presumably, having them mutated could help parasites rapidly adapt to the environment, which may partially explain the loss of resistance for some field isolates under long term *in vitro* culture (see Table S2). The SNP in PF3D7_1455300 has a high frequency in SEA population, especially e-GMS, and thus appears to be not in LD with K13 mutations. However, it cannot be found in African populations. This SNP could be fixed in the population due to selection and become a signature for the SEA population.

The SNP in PF3D7_0710400-475135 and deletions found in PF3D7_0710400-474979 and PF3D7_1368800 were only significantly associated with K13 in e-GMS. The regional differences observed between e-GMS and w-GMS may be partially related to the hard selective sweep that took place in the area, whereby a resistant C580Y lineage, which originated from Pailin, spread to northeastern Thailand and southern Laos, outcompeted other parasites, and dominated the area (Miotto et al., 2013, 2015; Zhu et al., 2018; Imwong et al., 2017). It was suggested that this C580Y lineage may possess higher fitness or transmission than other resistant parasites, which thus led to the dominance. In the analysis of the Pailin K13 population, where the K13 C580Y mutation became the only K13 mutation type in 2012, the percentage of K13 parasites possessing the EnDNArep genotype further increased to around 90%, indicating a close relationship between the EnDNArep genotype and the dominating C580Y lineage (Figure S5). The C580Y parasites from w-GMS were shown to have a different origin, explaining why these parasites have a different association pattern between the EnDNArep genotype and K13 mutations.

It is noteworthy that fully 50% of the DNA repair genes mutated preferentially in the resistant strains are genes that participate in homology-directed repair (HR), which is mostly error free. Resistant strains have mutations anticipated to reduce HR, which would shunt repair of DSBs to the error-guaranteed alternative nonhomologous end-joining pathway (Alt-NHEJ), which is expected to provide repair of DSBs in the absence of a homologous sequence (Kirkman et al., 2014). For Alt-NHEJ, the joining of microhomology on either side of the breakpoint necessitates an associated deletion or addition, leading to potential mutations (Kirkman et al., 2014, McVey and Lee, 2008). In addition, it is noteworthy that SSBs that are nearby on opposite strands can lead to DSBs. As the alkaline comet assay primarily detects SSBs (Olive and Banáth, 2006), the observation that resistant parasites have reduced levels of SSBs is consistent with a reduction in artemisinin-induced toxicity. Taken together, these parasites are expected to be simultaneously resistant to DNA damage-induced cytotoxicity while, at the same time, being prone to DNA damage-induced mutations.

We also observed the enhancement of DNA repair in the Dd2 parasite, albeit to a lower extent as compared with KH44 and the resistant parasite isolates (Figure S3). Dd2 was previously linked to the ARMD phenotype but is fully susceptible to artemisinin and has a WT K13 locus. It also possesses three out of seven mutations from the EnDNArep genotype (Table S1), indicating a potential link between the EnDNArep genotype and the accelerated development of drug resistance in general. As one of the ARMD strains, Dd2 was shown to have defects in sensing DNA damage and triggering DNA repair (Gupta et al., 2016) and, therefore, has a higher tolerance to damage, which led to higher mutation rates and accelerated development of resistance to multiple drugs (Castellini et al., 2011, Trotta et al., 2004). Although there have been debates on the existence of the ARMD phenotype in Southeast Asia, and although the ARMD phenotype was previously linked to mismatch repair pathways

CellPress
OPEN ACCESS

(Bopp et al., 2013, Claessens et al., 2014, Castellini et al., 2011), both previous studies (Lee and Fidock, 2016, Miotto et al., 2013) and evidence here have suggested potential links between mutations in DNA repair genes, accelerated rates of resistance development, and in-the-field artemisinin resistance development.

The data obtained here provide an attractive model linking DNA replication and artemisinin resistance; however, it is now important that this study is extended to include a larger number of clinical isolates from a wider geographic region. This would provide a better understanding on how the enhanced DNA repair phenotype is distributed in SEA and, more importantly, in Africa. Importantly, it would also provide us with a clearer picture on which genetic factors are more important for the phenotype and, therefore, make a more significant contribution. Ultimately, it will be necessary to experimentally validate these genetic associations and thereby definitively identify the critical mutations that underpin the DNA repair phenotype.

In conclusion, consistent with previous observations (Miotto et al., 2013, 2015; Zhu et al., 2018), the existence of parasites like KH44 indicated a founder population for drug resistance in the GMS with pre-deposing genetic factors, including mutations in DNA repair genes. Our study indicates that these mutations result in enhanced DNA repair that may enable parasites to tolerate more assaults on DNA; provide them with a higher survival chance; and, consequently, aids in the establishment and development of resistance. Future studies now need to further evaluate the importance of DNA damage repair in artemisinin resistance development, as this would, together with previous identified genetic factors (Miotto et al., 2013, 2015; Zhu et al., 2018), greatly aid in the identification and surveillance of areas that are particularly at risk to develop artemisinin resistance.

STAR★METHODS

Detailed methods are provided in the online version of this paper and include the following:

- KEY RESOURCES TABLE
- RESOURCE AVAILABILITY
  - Lead Contact
  - Materials Availability
  - Data and Code Availability
- EXPERIMENTAL MODEL AND SUBJECT DETAILS
- METHOD DETAILS
  - Treatment of purified plasmid DNA with activated artesunate
  - Fabrication of CometChip
  - Alkaline CometChip
  - Enrichment of live ring stage parasites using streptolysin O (SLO)
  - Enrichment of schizont stage parasites using Percoll gradient
  - On-chip treatment using hydrogen peroxide
  - Off-chip treatment using artesunate
  - SNPs analysis
- QUANTIFICATION AND STATISTICAL ANALYSIS

SUPPLEMENTAL INFORMATION

Supplemental Information can be found online at https://doi.org/10.1016/j.celrep.2020.107996.

ACKNOWLEDGMENTS

We would like to thank David Fidock (Columbia University, New York, NY, USA) for the *P. falciparum* Dd2 and Dd2^{I543T} strains. This work was supported by the National Research Foundation, Prime Minister's Office, Singapore, through the Singapore-MIT Alliance for Research and Technology (SMART) BioSystems and Micromechanics (BioSym) Interdisciplinary Research Group (IRG) (to A.X. and J.H.) and Antimicrobial Resistance IRG (to A.X., J.H., M.Z., W.C., and P.R.P.); and by the Bill and Melinda Gates Foundation (Global Health grant number OPP1040463 to A.X., C.W., P.P., G.X., and P.R.P.).

AUTHOR CONTRIBUTIONS

A.X., B.P.E., J.H., and P.R.P. conceptualized the study. A.X. and I.J.J.T. performed CometChip experiments. A.X. and M.C. performed K13 mutation verification on the *P. falciparum* Dd2 and Dd2^{I543T} strains. A.X., J.H., and P.R.P. performed the data analysis. P.P. and X.G. performed experiments on activated artesunate with purified DNA. A.X., C.J.W., B.P.E., J.H., and P.R.P drafted the manuscript, which was reviewed by all authors.

DECLARATION OF INTERESTS

B.P.E. is a co-inventor on a patent for the CometChip.

Received: December 31, 2019
Revised: May 21, 2020
Accepted: July 14, 2020
Published: August 4, 2020

REFERENCES

AlKadi, H.O. (2007). Antimalarial drug toxicity: a review. Chemotherapy *53*, 385–391.

Amaratunga, C., Witkowski, B., Khim, N., Menard, D., and Fairhurst, R.M. (2014). Artemisinin resistance in Plasmodium falciparum. Lancet Infect. Dis. *14*, 449–450.

Amaratunga, C., Lim, P., Suon, S., Sreng, S., Mao, S., Sopha, C., Sam, B., Dek, D., Try, V., Amato, R., et al. (2016). Dihydroartemisinin-piperaquine resistance in Plasmodium falciparum malaria in Cambodia: a multisite prospective cohort study. Lancet Infect. Dis. *16*, 357–365.

Ariey, F., Witkowski, B., Amaratunga, C., Beghain, J., Langlois, A.-C., Khim, N., Kim, S., Duru, V., Bouchier, C., Ma, L., et al. (2014). A molecular marker of artemisinin-resistant Plasmodium falciparum malaria. Nature *505*, 50–55.

Ashley, E.A., Dhorda, M., Fairhurst, R.M., Amaratunga, C., Lim, P., Suon, S., Sreng, S., Anderson, J.M., Mao, S., Sam, B., et al.; Tracking Resistance to Artemisinin Collaboration (TRAC) (2014). Spread of artemisinin resistance in Plasmodium falciparum malaria. N. Engl. J. Med. *371*, 411–423.

Bopp, S.E.R., Manary, M.J., Bright, A.T., Johnston, G.L., Dharia, N.V., Luna, F.L., McCormack, S., Plouffe, D., McNamara, C.W., Walker, J.R., et al. (2013). Mitotic evolution of Plasmodium falciparum shows a stable core genome but recombination in antigen families. PLoS Genet. *9*, e1003293.

Burrows, J. (2015). Microbiology: Malaria runs rings round artemisinin. Nature *520*, 628–630.

Castellini, M.A., Buguliskis, J.S., Casta, L.J., Butz, C.E., Clark, A.B., Kunkel, T.A., and Taraschi, T.F. (2011). Malaria drug resistance is associated with defective DNA mismatch repair. Mol. Biochem. Parasitol. *177*, 143–147.

Chaorattanakawee, S., Saunders, D.L., Sea, D., Chanarat, N., Yingyuen, K., Sundrakes, S., Saingam, P., Buathong, N., Sriwichai, S., Chann, S., et al. (2015). Ex Vivo Drug Susceptibility Testing and Molecular Profiling of Clinical Plasmodium falciparum Isolates from Cambodia from 2008 to 2013 Suggest

Emerging Piperaquine Resistance. Antimicrob. Agents Chemother. *59*, 4631–4643.

Cingolani, P., Platts, A., Wang, L., Coon, M., Nguyen, T., Wang, L., Land, S.J., Lu, X., and Ruden, D.M. (2012). A program for annotating and predicting the effects of single nucleotide polymorphisms, SnpEff: SNPs in the genome of Drosophila melanogaster strain w1118; iso-2; iso-3. Fly (Austin) *6*, 80–92.

Claessens, A., Hamilton, W.L., Kekre, M., Otto, T.D., Faizullabhoy, A., Rayner, J.C., and Kwiatkowski, D. (2014). Generation of antigenic diversity in Plasmodium falciparum by structured rearrangement of Var genes during mitosis. PLoS Genet. *10*, e1004812.

Danecek, P., Auton, A., Abecasis, G., Albers, C.A., Banks, E., DePristo, M.A., Handsaker, R.E., Lunter, G., Marth, G.T., Sherry, S.T., et al.; 1000 Genomes Project Analysis Group (2011). The variant call format and VCFtools. Bioinformatics *27*, 2156–2158.

Dondorp, A.M., Nosten, F., Yi, P., Das, D., Phyo, A.P., Tarning, J., Lwin, K.M., Ariey, F., Hanpithakpong, W., Lee, S.J., et al. (2009). Artemisinin resistance in Plasmodium falciparum malaria. N. Engl. J. Med. *361*, 455–467.

Dondorp, A.M., Yeung, S., White, L., Nguon, C., Day, N.P., Socheat, D., and von Seidlein, L. (2010). Artemisinin resistance: current status and scenarios for containment. Nat. Rev. Microbiol. *8*, 272–280.

Duru, V., Khim, N., Leang, R., Kim, S., Domergue, A., Kloeung, N., Ke, S., Chy, S., Eam, R., Khean, C., et al. (2015). Plasmodium falciparum dihydroartemisinin-piperaquine failures in Cambodia are associated with mutant K13 parasites presenting high survival rates in novel piperaquine in vitro assays: retrospective and prospective investigations. BMC Med. *13*, 305.

Ge, J., Wood, D.K., Weingeist, D.M., Bhatia, S.N., and Engelward, B.P. (2012). CometChip: single-cell microarray for high-throughput detection of DNA damage. In Methods in Cell Biology, P.M. Conn, ed. (Academic Press), pp. 247–268.

Ge, J., Prasongtanakij, S., Wood, D.K., Weingeist, D.M., Fessler, J., Navasummrit, P., Ruchirawat, M., and Engelward, B.P. (2014). CometChip: a high-throughput 96-well platform for measuring DNA damage in microarrayed human cells. J. Vis. Exp. (*92*), e50607.

Ge, J., Chow, D.N., Fessler, J.L., Weingeist, D.M., Wood, D.K., and Engelward, B.P. (2015). Micropatterned comet assay enables high throughput and sensitive DNA damage quantification. Mutagenesis *30*, 11–19.

Gil, J.P., Nogueira, F., Strömberg-Nörklit, J., Lindberg, J., Carrolo, M., Casimiro, C., Lopes, D., Arez, A.P., Cravo, P.V., and Rosário, V.E. (2003). Detection of atovaquone and Malarone resistance conferring mutations in Plasmodium falciparum cytochrome b gene (cytb). Mol. Cell. Probes *17*, 85–89.

Gopalakrishnan, A.M., and Kumar, N. (2015). Antimalarial action of artesunate involves DNA damage mediated by reactive oxygen species. Antimicrob. Agents Chemother. *59*, 317–325.

Gupta, D.K., Patra, A.T., Zhu, L., Gupta, A.P., and Bozdech, Z. (2016). DNA damage regulation and its role in drug-related phenotypes in the malaria parasites. Sci. Rep. *6*, 23603.

Imwong, M., Suwannasin, K., Kunasol, C., Sutawong, K., Mayxay, M., Rekol, H., Smithuis, F.M., Hlaing, T.M., Tun, K.M., van der Pluijm, R.W., et al. (2017). The spread of artemisinin-resistant Plasmodium falciparum in the Greater Mekong subregion: a molecular epidemiology observational study. Lancet Infect. Dis. *17*, 491–497.

Jackson, K.E., Spielmann, T., Hanssen, E., Adisa, A., Separovic, F., Dixon, M.W., Trenholme, K.R., Hawthorne, P.L., Gardiner, D.L., Gilberger, T., and Tilley, L. (2007). Selective permeabilization of the host cell membrane of Plasmodium falciparum-infected red blood cells with streptolysin O and equinatoxin II. Biochem. J. *403*, 167–175.

Kamau, E., Campino, S., Amenga-Etego, L., Drury, E., Ishengoma, D., Johnson, K., Mumba, D., Kekre, M., Yavo, W., Mead, D., et al. (2015). K13-propeller polymorphisms in Plasmodium falciparum parasites from sub-Saharan Africa. J. Infect. Dis. *211*, 1352–1355.

Kirkman, L.A., Lawrence, E.A., and Deitsch, K.W. (2014). Malaria parasites utilize both homologous recombination and alternative end joining pathways to maintain genome integrity. Nucleic Acids Res. *42*, 370–379.

Külzer, S., Bittl, V., and Przyborski, J.M. (2015). Fractionation of Plasmodium-infected human red blood cells to study protein trafficking. Methods Mol. Biol. *1270*, 71–80.

Kutner, S., Breuer, W.V., Ginsburg, H., Aley, S.B., and Cabantchik, Z.I. (1985). Characterization of permeation pathways in the plasma membrane of human erythrocytes infected with early stages of Plasmodium falciparum: association with parasite development. J. Cell. Physiol. *125*, 521–527.

Leang, R., Taylor, W.R., Bouth, D.M., Song, L., Tarning, J., Char, M.C., Kim, S., Witkowski, B., Duru, V., Domergue, A., et al. (2015). Evidence of Plasmodium falciparum Malaria Multidrug Resistance to Artemisinin and Piperaquine in Western Cambodia: Dihydroartemisinin-Piperaquine Open-Label Multicenter Clinical Assessment. Antimicrob. Agents Chemother. *59*, 4719–4726.

Lee, A.H., and Fidock, D.A. (2016). Evidence of a Mild Mutator Phenotype in Cambodian Plasmodium falciparum Malaria Parasites. PLoS ONE *11*, e0154166.

Lee, T.H., Guo, H., Wang, X., Kim, C., and Paterson, A.H. (2014). SNPhylo: a pipeline to construct a phylogenetic tree from huge SNP data. BMC Genomics *15*, 162.

Lu, F., Culleton, R., Zhang, M., Ramaprasad, A., von Seidlein, L., Zhou, H., Zhu, G., Tang, J., Liu, Y., Wang, W., et al. (2017). Emergence of Indigenous Artemisinin-Resistant Plasmodium falciparum in Africa. N. Engl. J. Med. *376*, 991–993.

Lundin, C., North, M., Erixon, K., Walters, K., Jenssen, D., Goldman, A.S.H., and Helleday, T. (2005). Methyl methanesulfonate (MMS) produces heat-labile DNA damage but no detectable in vivo DNA double-strand breaks. Nucleic Acids Res. *33*, 3799–3811.

McVey, M., and Lee, S.E. (2008). MMEJ repair of double-strand breaks (director's cut): deleted sequences and alternative endings. Trends Genet. *24*, 529–538.

Miotto, O., Almagro-Garcia, J., Manske, M., Macinnis, B., Campino, S., Rockett, K.A., Amaratunga, C., Lim, P., Suon, S., Sreng, S., et al. (2013). Multiple populations of artemisinin-resistant Plasmodium falciparum in Cambodia. Nat. Genet. *45*, 648–655.

Miotto, O., Amato, R., Ashley, E.A., MacInnis, B., Almagro-Garcia, J., Amaratunga, C., Lim, P., Mead, D., Oyola, S.O., Dhorda, M., et al. (2015). Genetic architecture of artemisinin-resistant Plasmodium falciparum. Nat. Genet. *47*, 226–234.

Mok, S., Ashley, E.A., Ferreira, P.E., Zhu, L., Lin, Z., Yeo, T., Chotivanich, K., Imwong, M., Pukrittayakamee, S., Dhorda, M., et al. (2015). Drug resistance. Population transcriptomics of human malaria parasites reveals the mechanism of artemisinin resistance. Science *347*, 431–435.

Mukherjee, A., Bopp, S., Magistrado, P., Wong, W., Daniels, R., Demas, A., Schaffner, S., Amaratunga, C., Lim, P., Dhorda, M., et al. (2017). Artemisinin resistance without pfkelch13 mutations in Plasmodium falciparum isolates from Cambodia. Malar. J. *16*, 195.

Olive, P.L., and Banáth, J.P. (2006). The comet assay: a method to measure DNA damage in individual cells. Nat. Protoc. *1*, 23–29.

Rasmussen, C., Nyunt, M.M., and Ringwald, P. (2017). Artemisinin-Resistant Plasmodium falciparum in Africa. N. Engl. J. Med. *377*, 305–306.

Russo, G., L'Episcopia, M., Menegon, M., Souza, S.S., Dongho, B.G.D., Vullo, V., Lucchi, N.W., and Severini, C. (2018). Dihydroartemisinin-piperaquine treatment failure in uncomplicated Plasmodium falciparum malaria case imported from Ethiopia. Infection *46*, 867–870.

Schneider, C.A., Rasband, W.S., Eliceiri, K.W., et al. (2012). NIH Image to ImageJ: 25 years of image analysis. Nat Methods *9*, 671–675.

Singh, N.P., McCoy, M.T., Tice, R.R., and Schneider, E.L. (1988). A simple technique for quantitation of low levels of DNA damage in individual cells. Exp. Cell Res. *175*, 184–191.

Sondén, K., Wyss, K., Jovel, I., Vieira da Silva, A., Pohanka, A., Asghar, M., Homann, M.V., Gustafsson, L.L., Hellgren, U., and Färnert, A. (2017). High Rate of Treatment Failures in Nonimmune Travelers Treated With Artemether-Lumefantrine for Uncomplicated Plasmodium falciparum Malaria in Sweden:

Retrospective Comparative Analysis of Effectiveness and Case Series. Clin. Infect. Dis. *64*, 199–206.

Straimer, J., Gnädig, N.F., Witkowski, B., Amaratunga, C., Duru, V., Ramadani, A.P., Dacheux, M., Khim, N., Zhang, L., Lam, S., et al. (2015). Drug resistance. K13-propeller mutations confer artemisinin resistance in Plasmodium falciparum clinical isolates. Science *347*, 428–431.

Strzyz, P. (2018). Concentrating on intrinsic disorder. Nat. Rev. Genet. *19*, 534.

Sutherland, C.J., Lansdell, P., Sanders, M., Muwanguzi, J., van Schalkwyk, D.A., Kaur, H., Nolder, D., Tucker, J., Bennett, H.M., Otto, T.D., et al. (2017). *pfk13*-Independent Treatment Failure in Four Imported Cases of Plasmodium falciparum Malaria Treated with Artemether-Lumefantrine in the United Kingdom. Antimicrob. Agents Chemother. *61*, e02382-16.

Sykora, P., Witt, K.L., Revanna, P., Smith-Roe, S.L., Dismukes, J., Lloyd, D.G., Engelward, B.P., and Sobol, R.W. (2018). Next generation high throughput DNA damage detection platform for genotoxic compound screening. Sci. Rep. *8*, 2771.

Taylor, S.M., Parobek, C.M., DeConti, D.K., Kayentao, K., Coulibaly, S.O., Greenwood, B.M., Tagbor, H., Williams, J., Bojang, K., Njie, F., et al. (2015). Absence of putative artemisinin resistance mutations among Plasmodium falciparum in Sub-Saharan Africa: a molecular epidemiologic study. J. Infect. Dis. *211*, 680–688.

Tilley, L., Straimer, J., Gnädig, N.F., Ralph, S.A., and Fidock, D.A. (2016). Artemisinin Action and Resistance in Plasmodium falciparum. Trends Parasitol. *32*, 682–696.

Trotta, R.F., Brown, M.L., Terrell, J.C., and Geyer, J.A. (2004). Defective DNA repair as a potential mechanism for the rapid development of drug resistance in Plasmodium falciparum. Biochemistry *43*, 4885–4891.

Van Hong, N., Amambua-Ngwa, A., Tuan, N.Q., Cuong, D., Giang, N.T., Van Dung, N., Tinh, T.T., Van Tien, N., Phuc, B.Q., Duong, T.T., et al. (2014). Severe malaria not responsive to artemisinin derivatives in man returning from Angola to Vietnam. Emerg. Infect. Dis. *20*, 1199–1202.

Weingeist, D.M., Ge, J., Wood, D.K., Mutamba, J.T., Huang, Q., Rowland, E.A., Yaffe, M.B., Floyd, S., and Engelward, B.P. (2013). Single-cell microarray enables high-throughput evaluation of DNA double-strand breaks and DNA repair inhibitors. Cell Cycle *12*, 907–915.

Witkowski, B., Amaratunga, C., Khim, N., Sreng, S., Chim, P., Kim, S., Lim, P., Mao, S., Sopha, C., Sam, B., et al. (2013). Novel phenotypic assays for the detection of artemisinin-resistant Plasmodium falciparum malaria in Cambodia: in-vitro and ex-vivo drug-response studies. Lancet Infect. Dis. *13*, 1043–1049.

Witkowski, B., Duru, V., Khim, N., Ross, L.S., Saintpierre, B., Beghain, J., Chy, S., Kim, S., Ke, S., Kloeung, N., et al. (2017). A surrogate marker of piperaquine-resistant Plasmodium falciparum malaria: a phenotype-genotype association study. Lancet Infect. Dis. *17*, 174–183.

Wood, D.K., Weingeist, D.M., Bhatia, S.N., and Engelward, B.P. (2010). Single cell trapping and DNA damage analysis using microwell arrays. Proc. Natl. Acad. Sci. USA *107*, 10008–10013.

World Health Organization (WHO) (2019). World malaria report 2019 (World Health Organization).

Wu, W.-M., Yao, Z.-J., Wu, Y.-L., Jiang, K., Wang, Y.-F., Cehn, H.-B., Shan, F., and Li, Y. (1996). Ferrous ion induced cleavage of the peroxy bond in qinghaosu and its derivatives and the DNA damage associated with this process. Chem. Commun. (*18*), 2213–2214.

Zhang, M., Wang, C., Otto, T.D., Oberstaller, J., Liao, X., Adapa, S.R., Udenze, K., Bronner, I.F., Casandra, D., Mayho, M., et al. (2018). Uncovering the essential genes of the human malaria parasite *Plasmodium falciparum* by saturation mutagenesis. Science *360*, eaap7847.

Zhu, L., Tripathi, J., Rocamora, F.M., Miotto, O., van der Pluijm, R., Voss, T.S., Mok, S., Kwiatkowski, D.P., Nosten, F., Day, N.P.J., et al.; Tracking Resistance to Artemisinin Collaboration I (2018). The origins of malaria artemisinin resistance defined by a genetic and transcriptomic background. Nat. Commun. *9*, 5158.

STAR★METHODS

KEY RESOURCES TABLE

REAGENT or RESOURCE	SOURCE	IDENTIFIER
Chemicals, Peptides, and Recombinant Proteins		
RPMI medium 1640	GIBCO	Cat#31800089
Sodium bicarbonate	Sigma-Aldrich	Cat#S5761
Hypoxanthine	Sigma-Aldrich	Cat#H9636
Gentamicin	GIBCO	Cat#15750060
D-sorbitol	Sigma-Aldrich	Cat#S6021
Giemsa stain	Sigma-Aldrich	Cat#GS500-500ML
AlbuMAX™ II Lipid-Rich BSA	GIBCO	Cat#11021037
Ferrous chloride	Sigma-Aldrich	Cat#372870-25G
Ferric chloride hexahydrate	Sigma-Aldrich	Cat#236489-5G
Deferoxamine	Sigma-Aldrich	Cat#D9533-1G
Ascorbic acid	Sigma-Aldrich	Cat#A92902-25G
Hemin	Sigma-Aldrich	Cat#51280-1G
Sodium hydroxide	Sigma-Aldrich	Cat#S5881-500G
Hydrogen peroxide	Merck	Cat#386790-100MLCN
Artesunate	Sigma-Aldrich	Cat#A3731
Low-melting point agarose	Invitrogen™	Cat#16520050
Phosphate buffered saline	Lonza	Cat#17-516F (38210090)
Sodium chloride	Sigma-Aldrich	Cat#S3014
Disodium EDTA	Sigma-Aldrich	Cat#E5134
Trizma® base	Sigma-Aldrich	Cat#T1503
Triton X-100	Sigma-Aldrich	Cat#T8787
Trizma® HCl	Sigma-Aldrich	Cat#T3523
SYBR Gold	Invitrogen™	Cat#S11494
Streptolysin O	Sigma-Aldrich	Cat#S5265-25KU
Dithiothreitol	Sigma-Aldrich	Cat#10197777001
Percoll	Sigma-Aldrich	Cat#P1644
Deposited Data		
KH strains VCF files	Wellcome Trust Sanger Institute, UK	Pf3K project release 5.1
CometChip raw data and analyzed results, Part one	This paper; Mendeley data	https://doi.org/10.17632/dfgj5ymxn7.1
CometChip raw data and analyzed results, Part two	This paper; Mendeley data	https://doi.org/10.17632/sj6fsjpcmp.1
CometChip raw data and analyzed results, Part three	This paper; Mendeley data	https://doi.org/10.17632/yncxdgwvs3.1
CometChip raw data and analyzed results, Part four	This paper; Mendeley data	https://doi.org/10.17632/dwcv62mrbn.1
CometChip raw data and analyzed results, Part five	This paper; Mendeley data	https://doi.org/10.17632/8gsnzy5ndj.1
CometChip raw data and analyzed results, Part six	This paper; Mendeley data	https://doi.org/10.17632/grdkg7rb83.1
Experimental Models: Organisms/Strains		
Plasmodium falciparum 3D7	MR4	N/A
Plasmodium falciparum Dd2 and Dd2^{I543T}	Straimer et al., 2015	N/A
Plasmodium falciparum KH004 clinical isolates	Ashley et al., 2014	N/A

(Continued on next page)

Continued

REAGENT or RESOURCE	SOURCE	IDENTIFIER
Recombinant DNA		
Purified DNA: Plasmid	Addgene	RRID: Addgene_128062
Software and Algorithms		
ImageJ	Schneider et al., 2012	https://imagej.nih.gov/ij/
MATLAB	The MathWorks Inc.	2018a
GATK tools	Broad Institute, USA	https://gatk.broadinstitute.org/hc/en-us
vcftools	Danecek et al., 2011	https://vcftools.github.io/index.html
SnpEff	Cingolani et al., 2012	http://snpeff.sourceforge.net/SnpEff.html
SNPhylo	Lee et al., 2014	https://github.com/thlee/SNPhylo
Guicometanalyzer	Wood et al., 2010	https://github.com/audreyx0206/MalariaCometChip
Other		
PDMS stamp	Wood et al., 2010	N/A
GelBond® film	Lonza	Cat#53748; 110 mm x 205 mm
Bio-One^{TM} 96-Well No Bottom Microplates	Greiner	Cat#07-000-626

RESOURCE AVAILABILITY

Lead Contact

Further information and requests for resources and reagents should be directed to and will be fulfilled by the Lead Contact, Peter R. Preiser (prpreiser@ntu.edu.sg).

Materials Availability

This study did not generate new unique reagents.

Data and Code Availability

The Guicometanalyzer is available at https://github.com/audreyx0206/MalariaCometChip (Wood et al., 2010). MalariaCometChip data generated and its related results are available at Mendeley data (DOI of Part I to VI: https://doi.org/10.17632/dfgj5ymxn7.1; https://doi.org/10.17632/sj6fsjpcmp.1; https://doi.org/10.17632/yncxdgwvs3.1; https://doi.org/10.17632/8gsnzy5ndj.1; https://doi.org/10.17632/grdkg7rb83.1).

EXPERIMENTAL MODEL AND SUBJECT DETAILS

P. falciparum strain 3D7 (MR4, USA) was cultured using fresh erythrocytes (Interstate Blood Bank, Inc) at 2.5% hematocrit in RPMI medium 1640 (GIBCO, USA) supplemented with 2.3 g/L sodium bicarbonate (Sigma-Aldrich, USA), 2.5 g/L AlbuMAX® (GIBCO, USA), 0.05 g/L of hypoxanthine (Sigma-Aldrich, USA) and 10 mg/L gentamicin (GIBCO, USA) (Noted as ‘complete RPMI’). Complete RPMI without AlbuMAX® supplemented was noted as ‘incomplete RPMI’. The parasites were incubated at 37°C with 5% CO_2, 3% O_2, and 92% N_2. Parasites culture were regularly synchronized using 5% D-sorbitol (Sigma-Aldrich, USA). Parasitemia was quantified by standard Giemsa based microscopy. In Giemsa based microscopy, blood smears were fixed and permeabilized using 100% methanol and stained with 1X Giemsa stain (Sigma-Aldrich, USA). Parasitemia was then manually estimated at 1000X magnification using light microscope Olympus IX63.

Dd2 and Dd2^{I543T} were obtained from Prof. David Fidock (Columbia University, USA), which were generated in the study published as “K13-propeller mutations confer artemisinin resistance in *Plasmodium falciparum* clinical isolates” (Straimer et al., 2015). KH004 serial clinical isolates were obtained from Dr. Charles J. Woodrow (Mahidol Oxford Tropical Medicine Research Unit, Thailand and the Centre for Tropical Medicine, UK), which were collected from Cambodia for the clinical trial entitled “A multicenter, randomized trial to detect *in vivo* resistance of *Plasmodium falciparum* to artesunate in patients with uncomplicated malaria (TRAC Study)” (Ashley et al., 2014). These field isolates were independently culture-adapted as reported by Mukherjee et al., 2017 (Mukherjee et al., 2017)., and all culture-adapted samples were confirmed to harbor monogenomic infections by molecular barcode analysis (Mukherjee et al., 2017). Cryo-preserved parasites were thawed using sorbitol gradient and cultured as described in previous section. Genotype information of the strains were obtained from Dr. Charles J. Woodrow and Prof. Olivo Miotto (Mahidol–Oxford Tropical Medicine Research Unit, Thailand and Nuffield Department of Medicine, the Medical Research Council Centre for Genomics and Global Health, University of Oxford and the Wellcome Trust Sanger Institute, Hinxton, UK).

METHOD DETAILS

Treatment of purified plasmid DNA with activated artesunate

Ferrous chloride ($FeCl_2$), ferric chloride hexahydrate ($FeCl_3.6H_2O$), deferoxamine (DFO, an iron chelator) and ascorbic acid (Vitamin C [Vc], the activator of hemin) were solubilised in deionised water and hemin was solubilised in 0.1 M sodium hydroxide (NaOH) (all from Sigma-Aldrich, USA). A 30 μl reaction mix was prepared for each treatment buffered with 20 mM Tris-HCl, pH8.0. ~500 ng purified plasmid DNA (~4 kb) was treated with different concentration of artesunate in presence and absence of 100 μM of ferric ion ($FeCl_3.6H_2O$), ferrous ion ($FeCl_2$) and DFO. Similarly, in the second experimental set up, DNA was treated with varying concentration of artesunate in presence of 100 μM of hemin and Vc. DFO and Vc were added prior to the addition of artesunate in their respective tubes, and DNA was lastly added to the tubes. 1 mM hydrogen peroxide (H_2O_2) was used as positive control to treat the plasmid (7.6 kb). Reaction mixtures were incubated for different time points at 37°C. After completion of incubation, 20 μl samples were run on 0.8% agarose gel and analyzed.

Fabrication of CometChip

CometChip assay was performed as described (Wood et al., 2010; Ge et al., 2012, 2014, 2015; Sykora et al., 2018; Weingeist et al., 2013). A homemade PDMS stamp with an array of micropillars was obtained from Professor Bevin Engelward (Department of Biomedical Engineering, Massachusetts Institute of Technology, USA). Briefly, 1% w/v agarose (BioRad, USA) was dissolved in phosphate-buffered saline (PBS) (Lonza, USA) and applied to the hydrophilic side of GelBond® film (Lonza, USA). The PDMS stamp was pressed on top of the molten agarose gel to generate arrays of microwells with around 40-50 μm in both diameter and depth, 240 μm space. The agarose gel was allowed to solidify for 15 min before the stamp was removed. A bottomless 96-well plate was applied on top of the agarose gel chip and secured by clips to form 96 macrowells, with an array of ~300 microwells in each.

Alkaline CometChip

For alkaline CometChip of *P. falciparum* parasites, 3% w/v agarose gel was used. Cell suspension was added to marcrowells of the chip and incubated at 37°C for 15 min in the incubator. Cells were captured in microwells by gravity and excess cells were washed off with PBS (Lonza, USA). After wash, the chip was overlaid with 1% w/v low-melting point agarose (ThermoFisher Scientific, USA) solution in PBS (melted and stabilized to 37°C before use). The chip was then kept at 4°C for 4 min for completely gelation of the overlaid agarose.

Alkaline lysis for the CometChip was performed at 4°C overnight. Alkaline lysis buffer (pH ~10) consisted of 2.5 M Sodium chloride, 100 mM disodium EDTA, 10 mM Trizma® base, and 1% v/v Triton X-100 (Sigma-Aldrich, USA) in deionized H_2O. After alkaline lysis, the chip was submerged in cold alkaline electrophoresis buffer (pH ~13.5), containing 0.3 M sodium hydroxide and 1 mM disodium EDTA, to unwind the nuclei at 4°C for 40min. Electrophoresis was then performed in the same buffer at 4°C for 30 min at 1 V/cm and ~300 mA. The chip was washed twice at room temperature with neutralization buffer containing 0.4 M Trizma® HCl (Sigma-Aldrich, USA). The CometChip was stained with 1X of SYBR Gold (Invitrogen, USA) for 30 min at room temperature in dark. Fluorescent images of the comets were captured at 40X magnification using epifluorescence microscopes, Nikon Eclipse 80i or Olympus IX83, with a 480-nm excitation filter. A detailed video describing CometChip can be found in study done by Ge et al. (Ge et al., 2014).

Enrichment of live ring stage parasites using streptolysin O (SLO)

25,000 units of Streptolysin O (Sigma-Aldrich, USA) was dissolved in 2.25 mL PBS (Lonza, USA) and stored at −80°C as stock solution. 1 M dithiothreitol (DTT) (Sigma-Aldrich, USA) was prepared in deionized water and stored at −20°C. Hemolytic unit (HU) was determined as described (Külzer et al., 2015). SLO was activated by mixing 1 M DTT with SLO stock solution at 1:10 ratio and incubating at room temperature for 15 min. 20 μL of compact RBCs were lysed with different concentrations of activated SLO for 6 min, and centrifuged. Absorbance at 412 nm wavelength of the supernatant was measured by infinite M200 pro plate reader (Tecan, Switzerland). The 1 HU is defined as the amount of SLO required to cause 50% lysis of RBCs and was determined for each batch of SLO. Live ring stage parasites were enriched as described previously (Jackson et al., 2007). Briefly, 0 to 18 h.p.i. ring stage culture with more than 10% parasitemia was lysed with 4 HUs of activated SLO and washed with 1X PBS until the supernatant turned colorless. The enriched parasites were resuspended with complete RPMI for further experiments.

Enrichment of schizont stage parasites using Percoll gradient

Parasites were synchronized at ring-stage (0-18 h.p.i) using 5% D-sorbitol and grow for another 30 h to achieve schizont stage. Schizont stage parasites (estimated 30-48 h.p.i) were purified using Percoll centrifugation as described (Kutner et al., 1985). Briefly, Percoll solution contains 63% Percoll (Sigma-Aldrich, USA), 27% 10X PBS and 10% incomplete RPMI. 1 mL parasite pellet were resuspended in 4ml incomplete RPMI. Resuspended parasites were gently layed on 10 mL Percoll gradient solution and underwent centrifugation at 2200 rpm, 11 min, brake 0. Schizont pellet were later recovered, washed with incomplete RPMI and proceed to further experiment.

On-chip treatment using hydrogen peroxide

Parasites enriched using SLO lysis or percoll purification were resuspended in complete RPMI at 0.5% v/v. 100 ul cell suspension was added to each macro-well. After LMPA was laid onto the chip, a new bottomless 96-well plate was applied onto the chip.

CellPress
OPEN ACCESS

Hydrogen peroxide solution, stored at 4°C and protected from light, was diluted with cold PBS and kept on ice. Cells were treated with 100 ul of different concentrations of hydrogen peroxide for 20 min at 4°C. The chip was submerged to cold alkaline lysis buffer immediately after treatment.

Off-chip treatment using artesunate

Artesunate (Sigma-Aldrich, USA) was dissolved to 50mM in 7.5% sodium bicarbonate solution (Sigma-Aldrich, USA). Aliquots of both were stored at −20°C and used one-time only without re-freezing. Parasites enriched using SLO lysis (0-18 h. p. i) or Percoll (30-48 h. p. i) purification were resuspended in complete RPMI at 0.5% v/v. 10X artesunate or mock solutions were prepared by diluting the stock with complete RPMI. Parasite suspension were incubated with different concentration of artesunate in 96-well plate for 1 h at 37°C. Treated parasites were transferred to CometChip by loading the parasite suspension into the macro-well of the CometChip using a multi-channel pipette and proceeded as describe previously. Drug is removed by washing the chip with 1X PBS. Each experiment was performed in technical triplicates and data shown were collected for biological triplicates.

SNPs analysis

VCF files containing SNP information of various parasite strains were obtained from open source Pf3K project release 5.1 (Wellcome Trust Sanger Institute, UK), processed using GATK tools (Broad Institute, USA) and vcftools (Danecek et al., 2011), annotated using SnpEff (Cingolani et al., 2012). Phylogenetic tree based on SNP profile was generated using SNPhylo (Lee et al., 2014). Thank Prof. Arjen Dondorp for providing these samples, and MalariaGEN and Prof. Dominic Kwiatkowski group for sequencing and making the data available.

QUANTIFICATION AND STATISTICAL ANALYSIS

MalariaCometChip images were analyzed using Guicometanalyzer, a customized software developed in MATLAB (The MathWorks Inc., USA) as described in detail in Figure S2 (Wood et al., 2010; Ge et al., 2015). Briefly, approximately 300 ‘comets’ were generated in each well. The head and tail of the ‘comet’ are automatically recognized by the algorithm. The fluorescent intensity is recorded and used to calculate the relative proportion of DNA in the comet tail to the whole comet to obtain %Tail DNA. Same process is performed for each comet. Median of % tail DNA in each macro-well was then calculated from results generated in Guicometanalyzer. All experiments were performed in technical triplicates and data of biological triplicates were collected and shown in means ± SEM in this work. For artesunate and MMS treatment, data were normalized by keeping means of each CometChip in the biological triplicates same. One-way or two-way ANOVA were performed and significance (p value) was reported by Prism Graphpad 6. For experiments where *P. falciparum* 3D7 was treated with hydrogen peroxide and *P. falciparum* 3D7, Dd2 and Dd2^{I543T} were treated with artesunate, ordinary one-way ANOVA was used and Dunnett’s multiple comparison tests were performed between vehicle control (0 μM) and respective concentrations. For Cambodian KH004 isolates treated with artesunate, two-way ANOVA was used, where comparisons were made between vehicle control and each concentration, and p values were adjusted after Šidák correction. Gene association were tested by Chi-square analysis and threshold of p value was adjusted after Bonferroni correction.

Cell Reports, Volume *32*

Supplemental Information

K13-Mediated Reduced Susceptibility to Artemisinin in *Plasmodium falciparum* Is Overlaid on a Trait of Enhanced DNA Damage Repair

Aoli Xiong, Prem Prakash, Xiaohong Gao, Marvin Chew, Ian Jun Jie Tay, Charles J. Woodrow, Bevin P. Engelward, Jongyoon Han, and Peter R. Preiser

Supplemental material

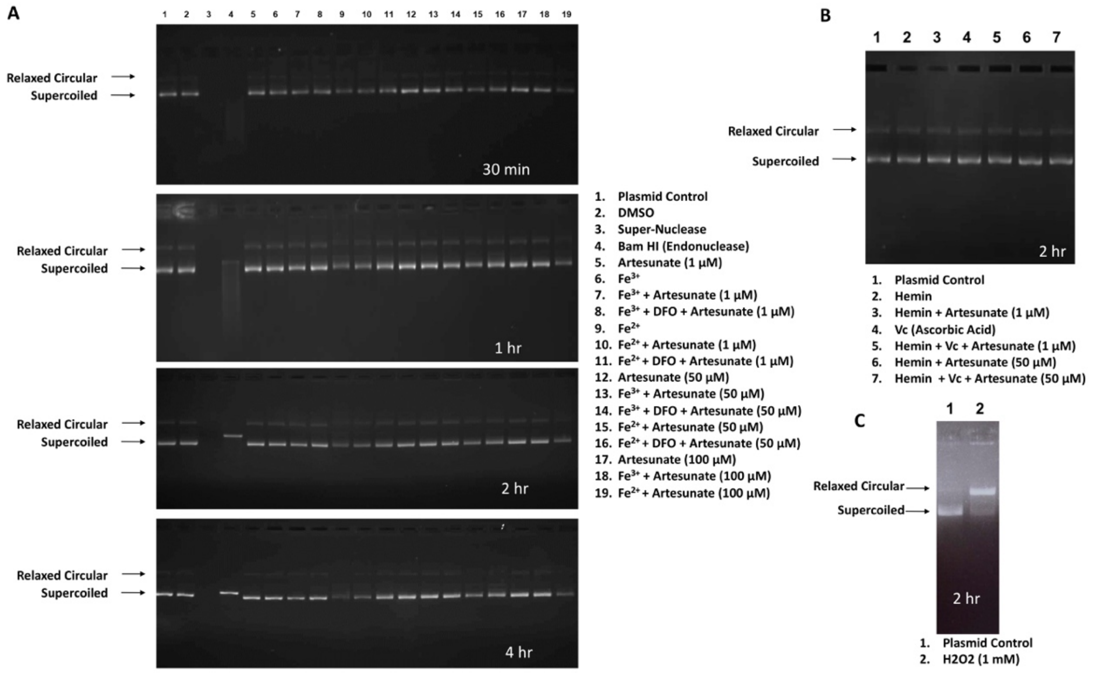

Supplementary figure 1. Activated artesunate does not induce DNA damage of purified DNA
A. Induced activation of artesunate by ferrous ion (Fe^{2+}) and its effect on purified plasmid DNA. B. Activation of artesunate by reduced hemin (haem) using ascorbic acid (Vc) and plasmid treatment. C. H_2O_2 used as positive control for plasmid DNA damage. Related to STAR Method.

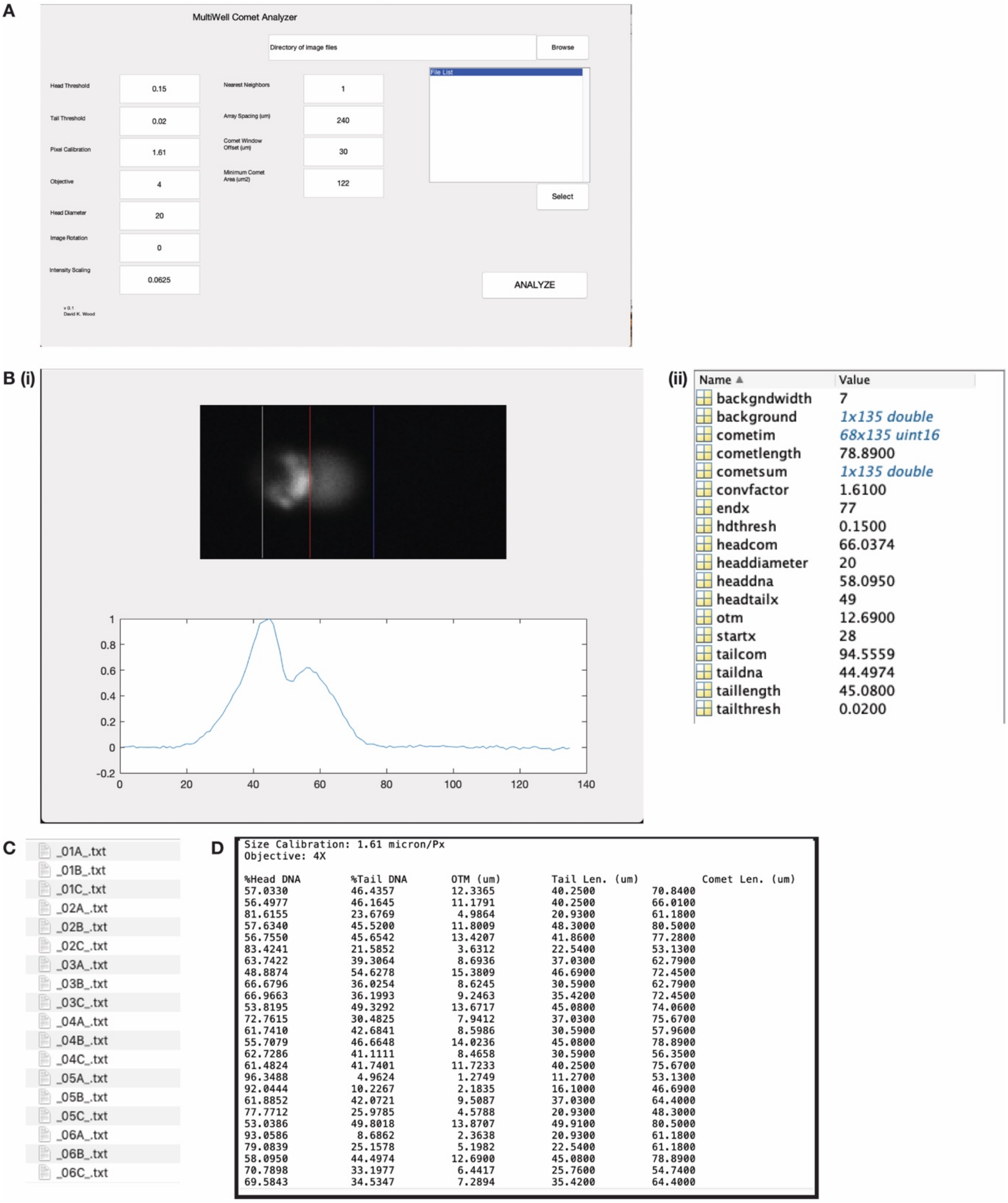

Supplementary figure 2. Illustration of image analysis using guicometanalyzer algorithm. Guicometanalyzer is a customized software developed in MATLAB that used to analyze images generated from CometChip experiment. A. Dialogue window for file selection and parameter setting. When running the software in MATLAB, the dialogue window would pop out, where parameters can be set or adjusted. Image files, labelled using the well position (i.e. 01A), can be then imported by browsing file direction. B. Interim result of individual comet. (i) Comet head (between while line and red line) and tail (between red line and blue line) recognized by the algorithm. (ii) Values reported by the algorithm. During the analysis, the algorithm can automatically identify comet, differentiate head and tail and report values accordingly as shown in the figure. C. Text file list generated by the algorithm for each macro-well. D. Data layout in each text file. For each image file representing one macro-well, a text file containing all the reported value will be generated. Median can then be calculated for each well and exported to excel file for further analysis. Related to STAR Method.

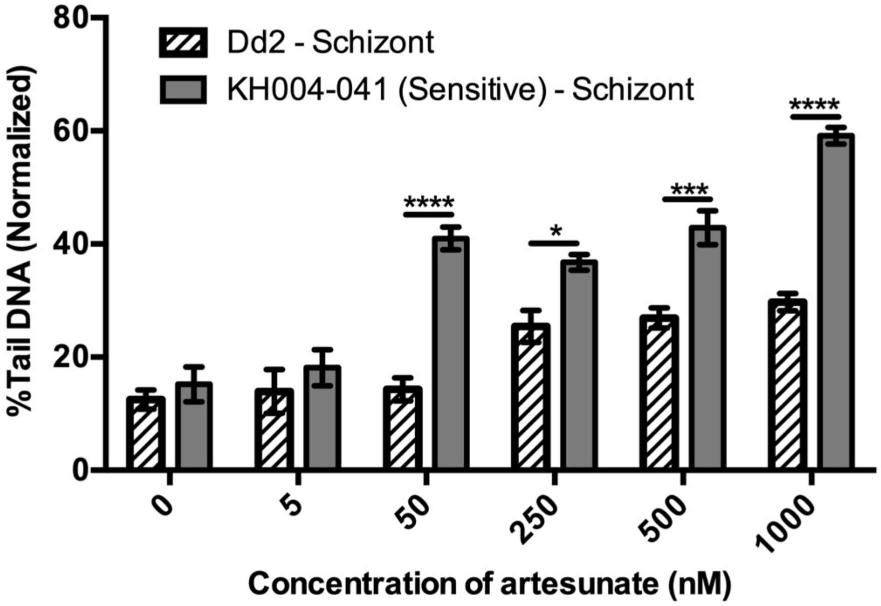

Supplementary figure 3. Comparison of artesunate-induced DNA damage level in Dd2 and Sensitive Cambodian isolate KH004-041.

Schizont stage parasites were treated with different concentration of artesunate for 1 hour and subjected to analysis on alkaline CometChip. Data was normalized within each treatment group. N≥3. *p<0.05, ***p<0.001, ****p<0.0001, two-way ANOVA. Comparison were made between Dd2 and KH004-041 at each concentration. Same data were plotted separately in Figure 2B and Figure 3A. Related to Figure 2 and Figure 3.

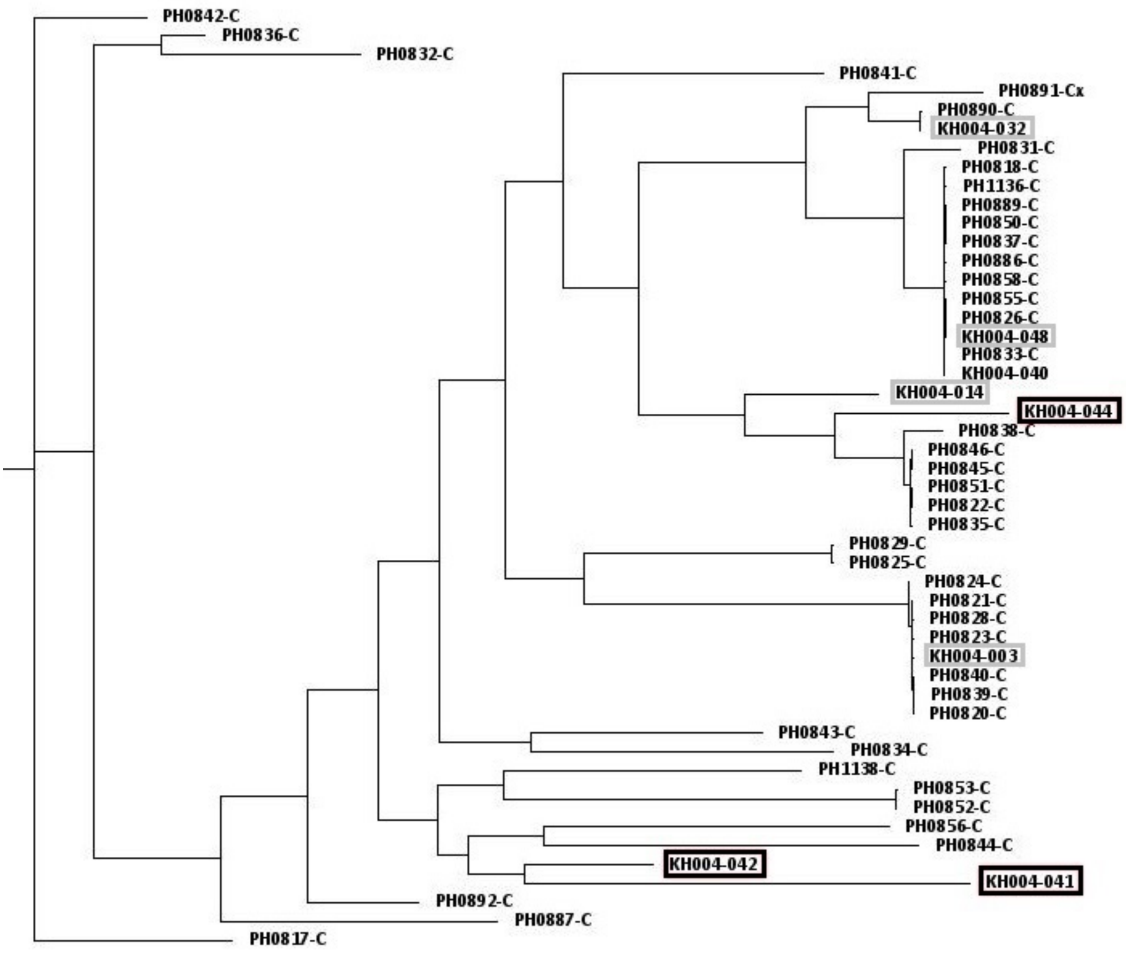

Supplementary figure 4. Phylogenetic tree based on SNP profile of samples from Pailin, Cambodia 2011.
VCF file containing SNPs across the whole genome were obtained from open source Pf3K project release 5.1 and analyzed as described in the Method. Black and grey box highlighted strains tested using CometChip. Among them, artemisinin sensitive isolates are labeled in black box, and artemisinin resistant isolates are labeled in grey box. Related to Figure 3 and STAR Method.

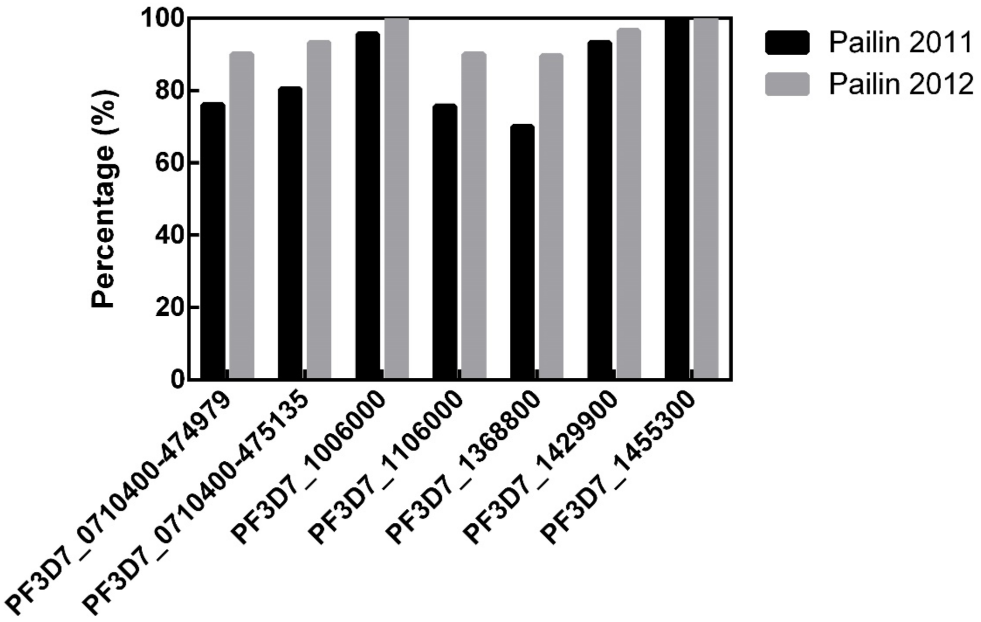

Supplementary figure 5. Temporal change of the frequency of the 'EnDNArep genotype' in K13 parasites from 2011 (black bar) to 2012 (grey bar) in Pailin, Cambodia.

Related to Figure 5.

Supplementary table 1. Distribution of mutations in 'EnDNArep genotype' in the KH and Dd2 strains. Artemisinin sensitive (red) and resistant (black) strains were compared. Mutant, heterozygote and wild type were labeled in orange, yellow and white, respectively. Related to table 1

Gene ID	Position	KH004 -041	KH004 -042	Dd2	KH004 -044	KH004 -003	KH004 -014	KH004 -040	KH004 -048	KH004 -032
PF3D7_0710400	474979									
	475135									
PF3D7_1006000	257175									
PF3D7_1106000	257027									
PF3D7_1368800	2731444									
PF3D7_1429900	1178944			TGA TAA TG	*	*	T	T	T	*
PF3D7_1455300	2260945									

Wild type

Heterozygote

Mutant

Supplementary table 2. List of affected genes. Related to Table 1.

Gene ID	Position	Product	ID of Affected gene(s)	Product description	Predicted function of affected gene(s)
PF3D7_0710400	474979 & 475135	DNA repair protein RAD14, putative	PF3D7_0710200	conserved Plasmodium protein, unknown function	GO:0005634 nucleus
			PF3D7_0710300	conserved protein, unknown function	GO:0016021 integral component of membrane; GO:0006810 transport; GO:0020011 apicoplast
			PF3D7_0710500	conserved protein, unknown function	GO:0020011 apicoplast
			PF3D7_0710600	60S ribosomal protein L34	GO:0005622 intracellular; GO:0005840 ribosome; GO:0003735 structural constituent of ribosome; GO:0006412 translation; GO:0022625 cytosolic large ribosomal subunit; GO:0005634 nucleus
PF3D7_1006000	257175	IMP1-like protein, putative	PF3D7_1005800	conserved Plasmodium membrane protein, unknown function	GO:0016021 integral component of membrane; GO:0005524 ATP binding; GO:0006810 transport
			PF3D7_1005900	conserved protein, unknown function	GO:0005737 cytoplasm; GO:0005524 ATP binding; GO:0016787 hydrolase activity; GO:0016740 transferase activity; GO:0020011 apicoplast
			PF3D7_1006100	CCR4-NOT transcription complex subunit 5, putative	GO:0005634 nucleus; GO:0006355 regulation of transcription, DNA-templated; GO:0030014 CCR4-NOT complex
			PF3D7_1006200	DNA/RNA-binding protein Alba 3	GO:0003676 nucleic acid binding; GO:1903561 extracellular vesicle; GO:0020020 food vacuole; GO:0005634 nucleus; GO:0003677 DNA binding; GO:0003723RNA binding; GO:0003729 mRNA binding; GO:0005515 protein binding
PF3D7_1106000	257027	RuvB-like helicase 2	PF3D7_1105800	conserved Apicomplexan protein, unknown function	GO:0020020 food vacuole; GO:0005739 mitochondrion; GO:0003729 mRNA binding
			PF3D7_1105900	conserved Plasmodium membrane protein, unknown function	GO:0016021 integral component of membrane; GO:0005886 plasma membrane; GO:0006810 transport

			PF3D7_1106100	apicoplast ribosomal protein S15 precursor, putative	GO:0005622 intracellular; GO:0005840 ribosome; GO:0003735 structural constituent of ribosome; GO:0006412 translation; GO:0020011 apicoplast
			PF3D7_1106200	conserved Plasmodium protein, unknown function	GO:0020011 apicoplast
PF3D7_1368800	2731444	DNA repair endonuclease XPF, putative	PF3D7_1368700	mitochondrial carrier protein, putative	GO:0022857 transmembrane transporter activity; GO:0055085 transmembrane transport; GO:0005743 mitochondrial inner membrane; GO:0006810 transport
PF3D7_1429900	1178944	ADP-dependent DNA helicase RecQ	PF3D7_1429800	coatomer subunit beta, putative	GO:0030126 COPI vesicle coat; GO:0030117 membrane coat GO:0005488 binding; GO:0005198 structural molecule activity; GO:0006886 intracellular protein transport; GO:0016192 vesicle-mediated transport; GO:0006888ER to Golgi vesicle-mediated transport
			PF3D7_1430000	conserved protein, unknown function	GO:0005737cytoplasm; GO:0016020 membrane
PF3D7_1455300	2260945	conserved Plasmodium protein	PF3D7_1455200	methyltransferase, putative, unspecified product	GO:0008168 methyltransferase activity; GO:0009234 menaquinone biosynthetic process; GO:0008152 metabolic process; GO:0006744 ubiquinone biosynthetic process
			PF3D7_1455400	hemolysin III	GO:0016021 integral component of membrane; GO:0020020 food vacuole; GO:0016020 membrane; GO:0044179 hemolysis in other organism

Supplementary table 3. List of known DNA repair genes. Related to Table 1.

Gene ID	Gene group	Gene function
PF3D7_0107800	GO	GO-0006281-BP-DNA repair
PF3D7_0107800	GO	GO-0006302-BP-double-strand break repair
PF3D7_0203300	GO	GO-0006289-BP-nucleotide-excision repair
PF3D7_0206000	GO	GO-0006281-BP-DNA repair
PF3D7_0217400	GO	GO-0006281-BP-DNA repair
PF3D7_0305600	GO	GO-0006281-BP-DNA repair
PF3D7_0408500	GO	GO-0006281-BP-DNA repair
PF3D7_0416400	GO	GO-0006281-BP-DNA repair
PF3D7_0505500	GO	GO-0006281-BP-DNA repair
PF3D7_0505500	GO	GO-0006298-BP-mismatch repair
PF3D7_0509500	GO	GO-0006281-BP-DNA repair
PF3D7_0513600	GO	GO-0006281-BP-DNA repair
PF3D7_0514100	GO	GO-0006281-BP-DNA repair
PF3D7_0605800	GO	GO-0006302-BP-double-strand break repair
PF3D7_0614800	GO	GO-0006284-BP-base-excision repair
PF3D7_0614800	GO	GO-0006281-BP-DNA repair
PF3D7_0619100	GO	GO-0006281-BP-DNA repair
PF3D7_0619100	GO	GO-0009380-CC-excinuclease repair complex
PF3D7_0628600	GO	GO-0006281-BP-DNA repair
PF3D7_0706700	GO	GO-0006298-BP-mismatch repair
PF3D7_0710100	GO	GO-0006281-BP-DNA repair
PF3D7_0710400	GO	GO-0006289-BP-nucleotide-excision repair
PF3D7_0725000	GO	GO-0006298-BP-mismatch repair
PF3D7_0726300	GO	GO-0006298-BP-mismatch repair
PF3D7_0803400	GO	GO-0000724-BP-double-strand break repair via homologous recombination
PF3D7_0910500	GO	GO-0006281-BP-DNA repair
PF3D7_0917100	GO	GO-0006284-BP-base-excision repair
PF3D7_0917100	GO	GO-0006289-BP-nucleotide-excision repair
PF3D7_0934100	GO	GO-0006289-BP-nucleotide-excision repair
PF3D7_1003700	GO	GO-0006281-BP-DNA repair
PF3D7_1006000	GO	GO-0006284-BP-base-excision repair
PF3D7_1008200	GO	GO-0006281-BP-DNA repair
PF3D7_1011700	GO	GO-0006289-BP-nucleotide-excision repair
PF3D7_1012000	GO	GO-0006281-BP-DNA repair
PF3D7_1037600	GO	GO-0006281-BP-DNA repair
PF3D7_1037600	GO	GO-0006289-BP-nucleotide-excision repair
PF3D7_1106000	GO	GO-0006281-BP-DNA repair
PF3D7_1107400	GO	GO-0006281-BP-DNA repair
PF3D7_1112600	GO	GO-0006281-BP-DNA repair
PF3D7_1117800	GO	GO-0006298-BP-mismatch repair
PF3D7_1129500	GO	GO-0006284-BP-base-excision repair
PF3D7_1140300	GO	GO-0006281-BP-DNA repair
PF3D7_1215700	GO	GO-0006298-BP-mismatch repair
PF3D7_1244200	GO	GO-0006281-BP-DNA repair
PF3D7_1250800	GO	GO-0006289-BP-nucleotide-excision repair
PF3D7_1303800	GO	GO-0006281-BP-DNA repair
PF3D7_1304100	GO	GO-0006281-BP-DNA repair
PF3D7_1314900	GO	GO-0006281-BP-DNA repair
PF3D7_1316900	GO	GO-0006281-BP-DNA repair
PF3D7_1332100	GO	GO-0006281-BP-DNA repair
PF3D7_1332600	GO	GO-0006281-BP-DNA repair
PF3D7_1338400	GO	GO-0006281-BP-DNA repair
PF3D7_1353500	GO	GO-0006281-BP-DNA repair
PF3D7_1368300	GO	GO-0006281-BP-DNA repair
PF3D7_1368800	GO	GO-0006281-BP-DNA repair
PF3D7_1405400	GO	GO-0006298-BP-mismatch repair

PF3D7_1415000	GO	GO-0006284-BP-base-excision repair
PF3D7_1427500	GO	GO-0006298-BP-mismatch repair
PF3D7_1429900	GO	GO-0006281-BP-DNA repair
PF3D7_1430600	GO	GO-0006281-BP-DNA repair
PF3D7_1432600	GO	GO-0006289-BP-nucleotide-excision repair
PF3D7_1441900	GO	GO-0006289-BP-nucleotide-excision repair
PF3D7_1455300	GO	GO-0006298-BP-mismatch repair
PF3D7_1467100	GO	GO-0006284-BP-base-excision repair
PF3D7_1471600	GO	GO-0000724-BP-double-strand break repair via homologous recombination
